# Immune dysregulation through longitudinal lymphocyte trajectories and their clinical determinants in hospitalized COVID-19 patients

**DOI:** 10.1186/s40635-026-00864-x

**Published:** 2026-02-06

**Authors:** José Pedro Cidade, Fabio Silvio Taccone, Luis Felipe Reyes, Laura Merson, Benjamin Lefevre, Barbara Wanjiru Citarella, Arie Zainul fatoni, Pedro Póvoa, the ISARIC Characterization Group, Luis Felipe Reyes, Laura Merson, Barbara Wanjiru Citarella, Sabriya Abdalasalam, Alaa Abdalfattah Abdalhadi, Naana Reyam Abdalla, Almthani Hamza Abdalrheem, Saedah Abdeewi, Esraa Hassan Abdelgaum, Mohamed Abdelhalim, Mohammed Abdelkabir, Sheryl Ann Abdukahil, Lamees Adil Abdulbaqi, Nurul Najmee Abdulkadir, Eman Abdulwahed, Rawad Abdunabi, Ryuzo Abe, Laurent Abel, Ahmed Mohammed Abodina, Amal Abrous, Kamal Abu Jabal, Nashat Abu Salah, Abdurraouf Abusalama, Subhash Acharya, Andrew Acker, Safia Adem, Manuella Ademnou, Neill K. J. Adhikari, Samuel Yaw Adu, Anthony Afum-Adjei Awuah, Melvin Agbogbatey, Saleh Al Ageel, Musaab Mohammed Ahmed, Aya Mustafa Ahmed, Zainab Ahmed Alaraji, Abdulrahman Ahmed Elhefnawy Enan, Reham Abdelhamid Ahmed Khalil, Ali Mostafa Ahmed Mohamed Abdelaziz, Kate Ainscough, Eka Airlangga, Tharwat Aisa, Ali Ait Hssain, Takako Akimoto, Ernita Akmal, Chika Akwani, Eman Al Qasim, Ahmed Alajeeli, Razi Alalqam, Zinah A. Alaraji, Safa Albatni, Angela Alberti, Tala Al-dabbous, Abdulkarim Aldoukali, Marta Alessi, Beatrice Alex, Kévin Alexandre, Abdulrahman Al-Fares, Asil Alflite, Huda Alfoudri, Qamrah Alhadad, Hoda Salem Alhaddad, Maali Khalid Mohamed Abdalla Alhasan, Hasan Alhouri, Ahmad Nabil Alhouri, Maha TagElser Mohammed Ali, Imran Ali, Yomna Ali Abdelghafar, Kazali Enagnon Alidjnou, Mahmoud Aljadi, Sarah Aljamal, Mohammed Alkahlout, Akram Alkaseek, Qabas Alkhafajee, Clotilde Allavena, Nathalie Allou, Abdulrahman Almjersah, Walaa Alrfaea, Moayad Alrifaee, Yousef Al-Sabaa, Entisar Alshareea, João Alves, João Melo Alves, Rita Alves, Joana Alves Cabrita, Maria Amaral, Amro Essam Amer, Nur Amira, Heidi Ammerlaan, Amos Amoako Adusei, John Amuasi, Roberto Andini, Claire Andrejak, Andrea Angheben, François Angoulvant, Séverine Ansart, Massimo Antonelli, Ardiyan Apriyana, Yaseen Arabi, Irene Aragao, Francisco Arancibia, Antonio Arcadipane, Patrick Archambault, Lukas Arenz, Jean-Benoît Arlet, Christel Arnold-Day, Lovkesh Arora, Elise Artaud-Macari, Diptesh Aryal, Motohiro Asaki, Angel Asensio, Elizabeth A. Ashley, Muhammad Ashraf, Muhammad Sheharyar Ashraf, Abir Ben Ashur, Franklin Asiedu-Bekoe, Namra Asif, Mohammad Asim, Grace Assi, Jean Baptiste Assie, Amirul Asyraf, Ahmed Atia, Minahel Atif, Asia Atif Abdelrhman Abdallahrs, Anika Atique, Moad Atlowly, Johann Auchabie, Hugues Aumaitre, Adrien Auvet, Ared Ayad, Ahmed Ayman Hassan Helmi, Laurène Azemar, Mohammed Azizeldin, Cecile Azoulay, Benjamin Bach, Antoine Bachelard, Delphine Bachelet, John Kenneth Baillie, J. Kevin Baird, Erica Bak, Agamemnon Bakakos, Nazreen Abu Bakar, Hibah Bileid Bakeer, Ashraf Bakri, Andriy Bal, Mohanaprasanth Balakrishnan, Alessandra Bandera, Firouzé Bani-Sadr, Nicholas Yuri Barbosa, Wendy S. Barclay, Saef Umar Barnett, Michaela Barnikel, Cleide Barrigoto, Marie Bartoli, Cheryl Bartone, Joaquín Baruch, Romain Basmaci, Muhammad Fadhli Hassin Basri, AbdAlkarim Batool, Denise Battaglini, Jules Bauer, Diego Fernando Bautista Rincon, Alexandra Bedossa, Ker Hong Bee, Husna Begum, Aleksandr Beljantsev, Beatriz Amorim Beltrão, Marine Beluze, Nicolas Benech, Lionel Eric Benjiman, Suzanne Bennett, Luís Bento, Jan-Erik Berdal, Delphine Bergeaud, José Luis Bernal Sobrino, Giulia Bertoli, Lorenzo Bertolino, Simon Bessis, Adam Betz, Sybille Bevilcaqua, Karine Bezulier, Amar Bhatt, Claudia Bianco, Sandra Bichoka, Farah Nadiah Bidin, Felwa Bin Humaid, Mohd Nazlin Bin Kamarudin, Muhannud Binnawara, Patrick Biston, Laurent Bitker, Pablo Blanco-Schweizer, Catherine Blier, Frank Bloos, Mathieu Blot, Filomena Boccia, Laetitia Bodenes, Debby Bogaert, Anne-Hélène Boivin, Ariel Bolanga, Isabela Bolaños, Pierre-Adrien Bolze, Aurelius Bonifasius, Joseph Bonney, Diogo Borges, Raphaël Borie, Elisabeth Botelho-Nevers, Lila Bouadma, Yasmine Bouaraba, Olivier Bouchaud, Sabelline Bouchez, Kévin Bouiller, Laurence Bouillet, Camile Bouisse, Latsaniphone Bountthasavong, Anne-Sophie Boureau, John Bourke, Maude Bouscambert, Aurore Bousquet, Marielle Boyer-Besseyre, Maria Boylan, Fernando Augusto Bozza, Axelle Braconnier, Luca Brazzi, Patrick Breen, David Brewster, Kathy Brickell, Tessa Broadley, Petra Bryda, Nina Buchtele, Polina Bugaeva, Marielle Buisson, Erlina Burhan, Donald Buri, Aidan Burrell, Ingrid G. Bustos, Denis Butnaru, André Cabie, Susana Cabral, Joana Cabrita, Eder Caceres, Cyril Cadoz, Rui Caetano Garcês, Mia Callahan, Jose Andres Calvache, João Camões, Paul Campbell, Josie Campisi, Cecilia Canepa, Mireia Cantero, Janice Caoili, Pauline Caraux-Paz, Filipa Cardoso, Sofia Cardoso, Nelson Cardoso, Filipe Cardoso, Simone Carelli, Nicolas Carlier, Thierry Carmoi, Gayle Carney, Inês Carqueja, Marie-Christine Carret, François Martin Carrier, Ida Carroll, Leonor Carvalho, Maire-Laure Casanova, Mariana Cascão, Siobhan Casey, José Casimiro, Bailey Cassandra, Silvia Castañeda, Guylaine Castor-Alexandre, Ana Catarino, François-Xavier Catherine, Roberto Cauda, Giulio- Giovanni Cavalli, Alexandros Cavayas, Adrian Ceccato, Ferruccio Ceriotti, Shelby Cerkovnik, Minerva Cervantes, Minerva Cervantes-Gonzalez, Muge Cevik, Anissa Chair, Catherine Chakveatze, Roberto Chalela, Adrienne Chan, Meera Chand, Jean-Marc Chapplain, Charlotte Charpentier, Julie Chas, Muhammad -Mobin Chaudry, Jonathan Samuel Chávez Iñiguez, Anjellica Chen, Matthew Pellan Cheng, Antoine Cheret, Thibault Chiarabini, Julian Chica, Suresh-Kumar Chidambaram, Leong Chin Tho, Catherine Chirouze, Hwa Jin Cho, Danoy Chommanam, Marie-Charlotte Chopin, Ting Soo Chow, Nathaniel Christy, Hiu Jian Chua, Jonathan Chua, José Miguel Cisneros Herreros, Anna Ciullo, Jennifer Clarke, Rolando Claure-Del Granado, Sara Clohisey, Peter David Coakley, Caitriona Cody, Megan Coles, Jennifer Coles, Gwenhaël Colin, Michael Collins, Pamela Combs, Jennifer Connolly, Marie Connor, Anne Conrad, Elaine Conway, Graham S. Cooke, Hugues Cordel, Amanda Corley, Sabine Cornelis, Arianne Joy Corpuz, Andrea Cortegiani, Grégory Corvaisier, Aoife Cotter, Sandrine Couffin-Cadiergues, Roxane Courtois, Stéphanie Cousse, Juthaporn Cowan, Rachel Cregan, Charles Crepy D Orleans, Cosimo Cristella, Gloria Crowl, Jonathan Crump, Claudina Cruz, Juan Luis Cruz Bermúdez, Jaime Cruz Rojo, Marc Csete, Ailbhe Cullen, Matthew Cummings, Gerard Curley, Elodie Curlier, Colleen Curran, Ana da Silva Filipe, Charlene da Silveira, Al-Awwab Dabaliz, Andrew Dagens, Darren Dahly, Peter Daley, Zaina Dalloul, Jo Dalton, Heidi Dalton, Seamus Daly, Juliana Damas, Joycelyn Dame, Cammandji Damien, Nick Daneman, Jorge Dantas, Menno de Jong, Fernando De La Calle Prieto, Gillian de Loughry, Diego de Mendoza, Etienne de Montmollin, Ana Isabel de Pinho Oliveira, Rosanna de Rosa, Thushan de Silva, Peter de Vries, Alexa Debard, Bianca DeBenedictis, Marie-Pierre Debray, Nathalie DeCastro, William Dechert, Romain Decours, Eve Defous, Isabelle Delacroix, Alexandre Delamou, Karen Delavigne, Nathalie M. Delfos, Andrea Dell Amore, Christelle Delmas, Pierre Delobel, Ismaila Deme, Elisa Demonchy, Emmanuelle Denis, Dominique Deplanque, Pieter Depuydt, Diane Descamps, Mathilde Desvallées, Santi Dewayanti, Pathik Dhangar, Alpha Diallo, Souleymane Taran Diallo, Sylvain Diamantis, Andrea Dias, André Dias, Juan Jose Diaz, Priscila Diaz, Rodrigo Diaz, Kévin Didier, Jean-Luc Diehl, Aurélien Dinh, Alphonsine Diouf, Yael Dishon, Cedric Djadda, Félix Djossou, Nikita Dobremel, Annemarie B. Docherty, Helen Doherty, Arjen M. Dondorp, Christl A. Donnelly, Yoann Donohue, Sean Donohue, Peter Doran, Céline Dorival, Eric D Ortenzio, Phouvieng Douangdala, James Joshua Douglas, Nathalie Dournon, Thomas Drake, Aoife Driscoll, Ibrahim Kwaku Duah, Vincent Dubee, François Dubos, Audrey Dubot-Pérès, Alexandre Ducancelle, Susanne Dudman, Abhijit Duggal, Paul Dunand, Jake Dunning, Mathilde Duplaix, Emanuele Durante-Mangoni, Lucian Durham, Bertrand Dussol, Xavier Duval, Anne Margarita Dyrhol-Riise, Sim-Choon Ean, Marco Echeverria-Villalobos, Michael Edelstein, Khadeja Ehzaz, Carla Eira, Mohammed El Sanharawi, Subbarao Elapavaluru, Mohammad Elbahnasawy, Brigitte Elharrar, Hamida ELMagrahi, Lauren Eloundou, Philippine Eloy, Tarek Elshazly, Wafa Elsokni, Aml Ahmed Eltayeb, Iqbal Elyazar, Zarief Kamel Emad, Hussein Embarek, Isabelle Enderle, Tomoyuki Endo, Gervais Eneli, Chan Chee Eng, Ilka Engelmann, Vincent Enouf, Olivier Epaulard, Haneen Esaadi, Mariano Esperatti, Catarina Espírito Santo, Marina Esposito-Farese, Rachel Essaka, João Estevão, Manuel Etienne, Anna Greti Everding, Mirjam Evers, Marc Fabre, Isabelle Fabre, Asgad Osman Abdalla Fadlalla, Amna Faheem, Arabella Fahy, Cameron J. Fairfield, Komal Fareed, Pedro Faria, Ahmed Farooq, Hanan Fateena, Salem Fatima, Arie Zainul Fatoni, Karine Faure, Raphaël Favory, Mohamed Fayed, Niamh Feely, Eoin Feeney, Susana Fernandes, Jorge Fernandes, Marília Andreia Fernandes, François-Xavier Ferrand, Joana Ferrão, Mário Ferraz, Isabel Ferreira, Sílvia Ferreira, Benigno Ferreira, Bernardo Ferreira, Nicolas Ferriere, Valentina Ferroni, Céline Ficko, Thomas Flament, Tom Fletcher, Aline-Marie Florence, Letizia Lucia Florio, Deirdre Flynn, Jean Foley, Victor Fomin, Patricia Fontela, Simon Forsyth, Denise Foster, Giuseppe Foti, Berline Fotso, Erwan Fourn, Robert A. Fowler, Marianne Fraher, Diego Franch-Llasat, Pierre Frange, John F. Fraser, Christophe Fraser, Ricardo Fritz, Stéphanie Fry, Nora Fuentes, G. Argin, Valérie Gaborieau, Rostane Gaci, Massimo Gagliardi, Jean-Charles Gagnard, Amandine Gagneux-Brunon, Sérgio Gaião, Linda Gail Skeie, Adham Mohamed Galal Mohamed Ramadan, Phil Gallagher, Carrol Gamble, Yasmin Gani, Arthur Garan, Rebekha Garcia, Noelia García Barrio, Julio Garcia Rodriguez, Julia Garcia-Diaz, Esteban Garcia-Gallo, Denis Garot, Valérie Garrait, Anatoliy Gavrylov, Alexandre Gaymard, Johannes Gebauer, Eva Geraud, Louis Gerbaud Morlaes, Nuno Germano, Malak Ghemmeid, Praveen Kumar Ghisulal, Jade Ghosn, Marco Giani, Carlo Giaquinto, Séverine Gibowski, Tristan Gigante, Guillermo Giordano, Michelle Girvan, Valérie Gissot, Gezy Giwangkancana, Daniel Glikman, Petr Glybochko, Eric Gnall, Geraldine Goco, François Goehringer, Siri Goepel, Jean-Christophe Goffard, Jin Yi Goh, Brigitta Golács, Jonathan Golob, Rui Gomes, Joan Gómez-Junyent, Marie Gominet, Alicia Gonzalez, Patricia Gordon, Isabelle Gorenne, Laure Goubert, Cécile Goujard, Tiphaine Goulenok, Margarite Grable, Jeronimo Graf, Edward Wilson Grandin, Pascal Granier, Giacomo Grasselli, Christopher A. Green, William Greenhalf, Segolène Greffe, Domenico Luca Grieco, Matthew Griffee, Fiona Griffiths, Ioana Grigoras, Albert Groenendijk, Fassou Mathias Grovogui, Heidi Gruner, Yusing Gu, Jérémie Guedj, Martin Guego, Anne-Marie Guerguerian, Daniela Guerreiro, Romain Guery, Anne Guillaumot, Laurent Guilleminault, Thomas Guimard, Daniel Haber, Ali Hachemi, Abdurrahman Haddud, Nadir Hadri, Wael Hafez, Fakhir Raza Haidri, Fatima Mhd Rida Hajij, Sheeba Hakak, Matthew Hall, Sophie Halpin, Shaher Hamdan, Abdelhafeez Hamdi, Ansley Hamer, Raph L. Hamers, Rebecca Hamidfar, Naomi Hammond, Terese Hammond, Lim Yuen Han, Matly Hanan, Rashan Haniffa, Kok Wei Hao, Hayley Hardwick, Ewen M. Harrison, Alan Hartman, Mohd Shahnaz Hasan, Mohammad Ali Nabil Hasan, Sulieman Hasan, Madiha Hashmi, Junaid Hashmi, Amoni Hassan, Ebtisam Hassanin, Muhammad Hayat, Ailbhe Hayes, Leanne Hays, Lars Heggelund, Ahmed Helmi, Ross Hendry, Martina Hennessy, Aquiles Rodrigo Henriquez-Trujillo, Maxime Hentzien, Diana Hernandez, Andrew Hershey, Astarini Hidayah, Eibhlin Higgins, Samuel Hinton, Hiroaki Hiraiwa, Maya Hites, Hikombo Hitoto, Yi Bin Ho, Antonia Ho, Alexandre Hoctin, Isabelle Hoffmann, Wei Han Hoh, Oscar Hoiting, Jan Cato Holter, Peter Horby, Juan Pablo Horcajada, Mabrouka Houderi, Stuart Houltham, Jimmy Ming-Yang Hsu, Jean-Sébastien Hulot, Abby Hurd, Iqbal Hussain, Aliae Mohamed Hussein, Mahmood Hussein, Fatima Ibrahim, Bashir Ibran, Samreen Ijaz, M. Arfan Ikram, Patrick Imbert, Rana Imran Sikander, Hugo Inácio, Carmen Infante Dominguez, Yun Sii Ing, Mariachiara Ippolito, Vera Irawany, Sarah Isgett, Tiago Isidoro, Nadiah Ismail, Margaux Isnard, Junji Itai, Danielle Jaafar, Salma Jaafoura, Hamza Jaber, Julien Jabot, Clare Jackson, Victoria Janes, Stéphane Jaureguiberry, Jeffrey Javidfar, Denise Jaworsky, Florence Jego, Anilawati Mat Jelani, Synne Jenum, Ruth Jimbo-Sotomayor, Ong Yiaw Joe, Ruth Noemí Jorge García, Cédric Joseph, Mark Joseph, Evelyn Joson, Mercé Jourdain, Philippe Jouvet, Anna Jung, Dafsah Juzar, Ouifiya Kafif, Florentia Kaguelidou, Neerusha Kaisbain, Sabrina Kali, Smaragdi Kalomoiri, Muhammad Aisar Ayadi Kamaluddin, Armand Saloun Kamano, Zul Amali Che Kamaruddin, Nadiah Kamarudin, Darshana Hewa Kandamby, Kong Yeow Kang, Dyah Kanyawati, Mohamed Karghul, Pratap Karpayah, Christiana Kartsonaki, Daisuke Kasugai, Kevin Katz, Christy Kay, Lamees Kayyali, Seán Keating, Aoife Kelly, Niamh Kelly, Sadie Kelly, Yvelynne Kelly, Maeve Kelsey, Sommay Keomany, Maeve Kernan, Younes Kerroumi, Sharma Keshav, Shams Khail, Sarah Khaled, Imrana Khalid, Zineb Khalil, Antoine Khalil, Irfan Khan, Sushil Khanal, Michelle E. Kho, Denisa Khoo, Saye Khoo, Ryan Khoo, Muhammad Nasir Khoso, Khor How Kiat, Yuri Kida, Peter Kiiza, Beathe Kiland Granerud, Anders Benjamin Kildal, Jae Burm Kim, Antoine Kimmoun, Paul Klenerman, Gry Kloumann Bekken, Stephen R. Knight, Robin Kobbe, Chamira Kodippily, Sabin Koirala, Stephanie Kouba, Karifa Kourouma, Mohamed Lamine Kourouma, Karolina Krawczyk, Ali Kredan, Vinothini Krishnan, Sudhir Krishnan, Oksana Kruglova, Anneli Krund, Pei Xuan Kuan, Ganesh Kumar, Deepali Kumar, Dinesh Kuriakose, Ethan Kurtzman, Demetrios Kutsogiannis, Galyna Kutsyna, Sylvie Kwedi, Konstantinos Kyriakoulis, Marie Lachatre, Karine Lacombe, Marie Lacoste, John G. Laffey, Nadhem Lafhej, Marie Lagrange, Fabrice Laine, Olivier Lairez, Antonio Lalueza, Marc Lambert, Marie Langelot-Richard, Vincent Langlois, Cédric Laouénan, Samira Laribi, Delphine Lariviere, Jamaica Laroza, Stéphane Lasry, Youssef Latifeh, Odile Launay, Didier Laureillard, Yoan Lavie-Badie, Andy Law, Teresa Lawrence, Minh Le, Clément Le Bihan, Cyril Le Bris, Georges Le Falher, Lucie Le Fevre, Quentin Le Hingrat, Marion Le Maréchal, Soizic Le Mestre, Guillaume Le Meut, Gwenaël Le Moal, Vincent Le Moing, Hervé Le Nagard, Ema Leal, Marta Leal Santos, Yi Lin Lee, Heng Gee Lee, James Lee, Biing Horng Lee, Todd C. Lee, Gary Leeming, Bénédicte Lefebvre, Laurent Lefebvre, Benjamin Lefèvre, Sylvie LeGac, Merili-Helen Lehiste, Jean-Daniel Lelievre, François Lellouche, Adrien Lemaignen, Véronique Lemee, Anthony Lemeur, Gretchen Lemmink, Ha Sha Lene, Jenny Lennon, Marc Leone, Tanel Lepik, Quentin Lepiller, François-Xavier Lescure, Mathieu Lesouhaitier, Andrew Letizia, Sophie Letrou, Bruno Levy, Yves Levy, Claire Levy-Marchal, Katarzyna Lewandowska, Gianluigi Li Bassi, Janet Liang, Geoffrey Liegeon, Wei Shen Lim, Kah Chuan Lim, Chantre Lima, Lim Lina, Bruno Lina, Andreas Lind, Guillaume Lingas, Sylvie Lion-Daolio, Keibun Liu, Marine Livrozet, Patricia Lizotte, Antonio Loforte, Navy Lolong, Leong Chee Loon, Diogo Lopes, Dalia Lopez-Colon, Jose W. López-Revilla, Anthony L. Loschner, Paul Loubet, Bouchra Loufti, Guillame Louis, Silvia Lourenco, Lee Lee Low, Jia Shyi Loy, Carlos Lumbreras Bermejo, Carlos M. Luna, Olguta Lungu, Miles Lunn, Liem Luong, Nestor Luque, Dominique Luton, Olavi Maasikas, Moïse Machado, Sara Machado, Gabriel Macheda, Claire Madelaine, Guillermo Maestro de la Calle, Rafael Mahieu, Sophie Mahy, Ana Raquel Maia, Lars S. Maier, Oumou Maiga Ascofare, Mylène Maillet, Nimisha Abdul Majeed, Maximilian Malfertheiner, Nadia Malik, Wajeeha Malik, Dayana Malla, Paddy Mallon, Fernando Maltez, Denis Malvy, Victoria Manda, Laurent Mandelbrot, Frank Manetta, Julie Mankikian, Edmund Manning, Aldric Manuel, Veronika Maráczi, Samuel Markowicz, Ana Marques, Megan Marshal, John Marshall, Dori-Ann Martin, Emily Martin, Guillaume Martin-Blondel, F. Eduardo Martinez, Ignacio Martin-Loeches, Martin Martinot, Alejandro Martín-Quiros, João Martins, Nuno Martins, Ana Martins, Gennaro Martucci, Olga Martynenko, Eva Miranda Marwali, Marsilla Marzukie, David Maslove, Sabina Mason, Sobia Masood, Moise Massoma, Palmer Masumbe, Mohd Basri Mat Nor, Moshe Matan, Christina Mathew, Mathieu Mattei, Laurence Maulin, Juergen May, Mayfong Mayxay, Thierry Mazzoni, Lisa Mc Sweeney, Colin McArthur, Peter McCanny, Aine McCarthy, Colin McCloskey, Rachael McConnochie, Sherry McDermott, Sarah E. McDonald, Natalie McEvoy, Allison McGeer, Kenneth A. McLean, Paul McNally, Bairbre McNicholas, Edel Meaney, Cécile Mear-Passard, Maggie Mechlin, Omar Mehkri, Ferruccio Mele, João João Mendes, Kusum Menon, France Mentré, Alexander J. Mentzer, Emmanuelle Mercier, Antoine Merckx, Mayka Mergeay-Fabre, António Mesquita, Osama Metwally, Agnès Meybeck, Dan Meyer, Mehdi Mezidi, Isabelle Michelet, Efstathia Mihelis, Vladislav Mihnovit, Duha Milad Abdullah, Jennene Miller, Hugo Miranda-Maldonado, Nor Arisah Misnan, Tahira Jamal Mohamed, Nouralsabah Mohamed, Nik Nur Eliza Mohamed, Alaa Mohamed Ads, Ahmed Reda Mohamed Elsayed Abdelhalim, Shrouk Fawze Mohammed Mostafa, Omer Abdullah Mohammedelhassan, Saad A. Moharam, Diana Molino, Elena Molinos, Brenda Molloy, Geraldine Moloney, Mary Mone, Agostinho Monteiro, Claudia Montes, Giorgia Montrucchio, Sarah Moore, Shona C. Moore, Lina Morales Cely, Marwa Morgom, Lucia Moro, Ana Motos, Clara Mouton Perrot, Julien Moyet, Suleiman Haitham Mualla, Aisha Kalsoom Mufti, Ng Yong Muh, Mo nes Muhaisen, Dzawani Muhamad, Jimmy Mullaert, Karl Erik Müller, Fredrik Müller, Daniel Munblit, Syed Muneeb Ali, Laveena Munshi, Aisling Murphy, Patrick Murray, Marlène Murris, Srinivas Murthy, Himed Musaab, Dana Mustafa, Dimitra Melia Myrodia, Farah Nadia Mohd-Hanafiah, Dave Nagpal, Blanka Nagybányai-Nagy, Herwin Nanda Boudoin, Mangala Narasimhan, Adel Gerges Nassif Metri, Nadège Neant, Coca Necsoi, Nikita Nekliudov, Matthew Nelder, Erni Juwita Nelwan, Emily Neumann, Wing Yiu Ng, Pauline Yeung Ng, Anthony Nghi, Duc Nguyen, Orna Ni Choileain, Niamh Ni Leathlobhair, Nerissa Niba, Alistair D. Nichol, Nurul Amani Mohd Noordin, Nurul Faten Izzati Norharizam, Mahdad Noursadeghi, Adam Nowinski, Saad Nseir, Leonard Numfor, Nurnaningsih Nurnaningsih, Dwi Utomo Nusantara, Elsa Nyamankolly, Fionnuala O. Brien, Annmarie O. Callaghan, Annmarie O Callaghan, Giovanna Occhipinti, Sarah O Connell, Derbrenn OConnor, Max O Donnell, Ebenezer Oduro-Mensah, Tawnya Ogston, Takayuki Ogura, Tak-Hyuk Oh, Sophie O Halloran, Katie O Hearn, Sally-Ann Ohene, Shinichiro Ohshimo, Agnieszka Oldakowska, João Oliveira, Joseph Oliver-Commey, Piero L. Olliaro, Inge Christoffer Olsen, Alsarrah Ali Mohammed Omer, Pierre Ondobo, David S. Y Ong, Jee Yan Ong, Wilna Oosthuyzen, Peter Openshaw, Saijad Orakzai, Claudia Milena Orozco-Chamorro, Mohamed Osama Elsayed Soliman, Linda O Shea, Miriam O Sullivan, Siti Zubaidah Othman, Eman Othman, Rachida Ouissa, Christian Owoo, Micheal Owusu, Ama Akyampomaa Owusu-Asare, Eric Oziol, Patricia Pacheco, Maïder Pagadoy, Justine Pages, Massimo Palmarini, Carlo Palmieri, Giovanna Panarello, Prasan Kumar Panda, Hem Paneru, Lai Hui Pang, Mauro Panigada, Nathalie Pansu, Aurélie Papadopoulos, Rachael Parke, Melissa Parker, Bruno Pastene, Fabian Patauner, Mohan Dass Pathmanathan, Luís Patrão, Patricia Patricio, Lisa Patterson, Christelle Paul, Mical Paul, Jorge Paulos, William A. Paxton, Jean-François Payen, Sandra L. Peake, Kalaiarasu Peariasamy, Miguel Pedrera Jiménez, Giles J. Peek, Florent Peelman, Nathan Peiffer-Smadja, Vincent Peigne, Mare Pejkovska, Paolo Pelosi, Rui Pereira, Daniel Perez, Thomas Perpoint, Antonio Pesenti, Lenina Pessey, Vincent Pestre, Lenka Petrou, Michele Petrovic, Ventzislava Petrov-Sanchez, Gilles Peytavin, Richard Odame Philips, Ooyanong Phonemixay, Soulichanya Phoutthavong, Michael Piagnerelli, Gilles Pialoux, Olivier Picone, Maria de Piero, Carlos Pimentel, Raquel Pinto, Catarina Pires, Lionel Piroth, Ayodhia Pitaloka, Chiara Piubelli, Riinu Pius, Simone Piva, Laurent Plantier, Hon Shen Png, Julien Poissy, Ryadh Pokeerbux, Maria Pokorska-Spiewak, Sergio Poli, Georgios Pollakis, Diane Ponscarme, Jolanta Popielska, Diego Bastos Porto, Andra-Maris Post, Douwe F. Postma, Valérie Pourcher, Diana Póvoas, Jeff Powis, Sofia Prapa, Viladeth Praphasiri, Sébastien Preau, Christian Prebensen, Jean-Charles Preiser, Anton Prinssen, Gamage Dona Dilanthi Priyadarshani, Lucia Proença, Sravya Pudota, Bambang Pujo Semedi, Peter Puplampu, Gregory Purcell, Luisa Quesada, Víctor Quirós González, Else Quist-Paulsen, Mohammed Quraishi, Fadi Qutishat, Christian Rabaud, Ebenezer Rabindrarajan, Aldo Rafael, Marie Rafiq, Abdelrahman Ragab, Mutia Rahardjani, Rozanah Abd Rahman, Ahmad Kashfi Haji Ab Rahman, Fernando Rainieri, Giri Shan Rajahram, Pratheema Ramachandran, José Ramalho, Ahmad Afiq Ramli, Grazielle Viana Ramos, Muhammad Asim Rana, Rajavardhan Rangappa, Hervé Raoul, Christophe Rapp, Thalha Rashan, Aasiyah Rashan, Ghulam Rasheed, Menaldi Rasmin, Indrek Rätsep, Cornelius Rau, Tharmini Ravi, Andre Real, Stanislas Rebaudet, Sarah Redl, Brenda Reeve, Dag Henrik Reikvam, Renato Reis, Jonathan Remppis, Hongru Ren, Hanna Renk, Anne-Sophie Resseguier, Matthieu Revest, Oleksa Rewa, Maria Ines Ribeiro, Denise Richardson, David Richardson, Laurent Richier, Siti Nurul Atikah Ahmad Ridzuan, Asgar Rishu, Patrick Rispal, Karine Risso, Maria Angelica Rivera Nuñez, Chiara Robba, André Roberto, David L. Robertson, Olivier Robineau, Ferran Roche-Campo, Paola Rodari, Simão Rodeia, Bernhard Roessler, Claire Roger, Amanda Rojek, Roberto Roncon-Albuquerque, Mélanie Roriz, Manuel Rosa-Calatrava, Michael Rose, Dorothea Rosenberger, Andrea Rossanese, Matteo Rossetti, Patrick Rossignol, Carine Roy, Benoît Roze, Desy Rusmawatiningtyas, Clark D. Russell, Maeve Ryan, Aleksander Rygh Holten, Isabela Saba, Sairah Sadaf, Musharaf Sadat, Valla Sahraei, Abdurraouf Said, Fodé Bangaly Sako, Moamen Salah, Ali Alaa Salah Eldin Mohamed Abbas, Nawal Salahuddin, Leonardo Salazar, Mohammed Saleh Alyasiri, Gabriele Sales, Charlotte Salmon Gandonniere, Hélène Salvator, Yehia Samir Shaaban Aly Orabi, Olivier Sanchez, Emely Sanchez, Angel Sanchez-Miralles, Gyan Sandhu, Zulfiqar Sandhu, Pierre-François Sandrine, Oana Săndulescu, Marlene Santos, Lurdes Santos, Shirley Sarfo-Mensah, Bruno Sarmento Banheiro, Benjamine Sarton, Sree Satyapriya, Rumaisah Satyawati, Yen Tsen Saw, Justin Schaffer, Tjard Schermer, Arnaud Scherpereel, Marion Schneider, János Schnur, Michael Schwameis, Gary Schwartz, Janet T. Scott, Nicholas Sedillot, Tamara Seitz, Mageswari Selvarajoo, Malcolm G. Semple, Rasidah B. T. Senian, Eric Senneville, Claudia Sepulveda, Tânia Sequeira, Ary Serpa Neto, Pablo Serrano Balazote, Ellen Shadowitz, Syamin Asyraf Shahidan, Laila Shalabi, Haitam Shames, Anuraj Shankar, Shaikh Sharjeel, Pratima Sharma, Catherine A. Shaw, Victoria Shaw, John Robert Sheenan, Rajesh Mohan Shetty, Mohiuddin Shiekh, Nobuaki Shime, Keiki Shimizu, Hiroaki Shimizu, Sally Shrapnel, Hoi Ping Shum, Nassima Si Mohammed, Ng Yong Siang, Jeanne Sibiude, Bountoy Sibounheuang, Louise Sigfrid, Piret Sillaots, Maria Joao Silva, Rogério Silva, Catarina Silva, Benedict Sim Lim Heng, Wai Ching Sin, Punam Singh, Mahendra Singh, Pompini Agustina Sitompul, Karisha Sivam, Vegard Skogen, Benjamin Smood, Coilin Smyth, Dominic So, Tze Vee Soh, Tom Solomon, Joshua Solomon, Emily Somers, Agnès Sommet, Rima Song, Tae Song, Jack Song Chia, Albert Sotto, Edouard Soum, Ana Chora Sousa, Marta Sousa, Maria Sousa Uva, Vicente Souza-Dantas, Mamadou Saliou Sow, Alexandra Sperry, Elisabetta Spinuzza, B. P. Sanka Ruwan Sri Darshana, Shiranee Sriskandan, Sarah Stabler, Thomas Staudinger, Stephanie-Susanne Stecher, Trude Steinsvik, Ymkje Stienstra, Birgitte Stiksrud, Eva Stolz, Amy Stone, Adrian Streinu-Cercel, Anca Streinu-Cercel, David Stuart, Decy Subekti, Jacky Y. Suen, Prasanth Sukumar, Charlotte Summers, Dubravka Supic, Deepashankari Suppiah, Magdalena Surovcová, Atie Suwarti, Andrey Svistunov, Sarah Syahrin, Augustina Sylverken, Konstantinos Syrigos, Shirin Tabrizi, Fabio S. Taccone, Shahdattul Mawarni Taib, Ewa Talarek, Sara Taleb, Cheikh Talla, Jelmer Talsma, Maria Lawrensia Tampubolon, Yan Chyi Tan, Kim Keat Tan, Taku Tanaka, Huda Taqdees, Coralie Tardivon, Yousef Tarek Kamal Mostafa, Ali Tarhabat, Pierre Tattevin, M. Azhari Taufik, Hassan Tawfik, Tze Yuan Tee, João Teixeira, Sze Kye Teoh, Vanessa Teotonio, François Téoulé, Olivier Terrier, Hubert Tessier-Grenier, Adrian Tey, Alif Adlan Mohd Thabit, Zhang Duan Tham, Suvintheran Thangavelu, Elmi Theron, Vincent Thibault, Simon-Djamel Thiberville, Benoît Thill, Jananee Thirumanickam, Shaun Thompson, Emma C. Thomson, David Thomson, Mathew Thorpe, Surain Raaj ThangaThurai, Ryan Thwaites, Paul Tierney, Vadim Tieroshyn, Peter S. Timashev, Jean-François Timsit, Noémie Tissot, Jordan Zhien Yang Toh, Maria Toki, Kristian Tonby, Sia Loong Tonnii, Marta Torre, Antoni Torres, Margarida Torres, Rosario Maria Torres Santos-Olmo, Hernando Torres-Zevallos, Aboubacar Tounkara, Michael Towers, Fodé Amara Traoré, Tony Trapani, Cécile Tromeur, Ioannis Trontzas, Jeanne Truong, Christelle Tual, Sarah Tubiana, Helen Tuite, Alexis F. Turgeon, Lance C. W. Turtle, Anders Tveita, Pawel Twardowski, Makoto Uchiyama, P. G. Ishara Udayanga, Andrew Udy, Roman Ullrich, Alberto Uribe, Asad Usman, Timothy M. Uyeki, Cristinava Vajdovics, Luís Val-Flores, Marcel van den Berge, Machteld van der Feltz, Peter Van der Voort, Sylvie Van Der Werf, Eric van Gorp, Laura van Gulik, Jarne van Hattem, Carolien van Netten, Frank van Someren Gréve, Gitte van Twillert, Hugo van Willigen, Noémie Vanel, Michael Varrone, Shoban Raj Vasudayan, Charline Vauchy, Shaminee Veeran, Aurélie Veislinger, Sara Ventura, Annelies Verbon, César Vieira, Deepak Vijayan, Judit Villar, Andrea Villoldo, Manivanh Vongsouvath, Fanny Vuotto, Suhaila Abdul Wahab, Noor Hidayu Wahab, Nadirah Abdul Wahid, Marina Wainstein, Laura Walsh, Steve Webb, Tan Pei Wen, Sanne Wesselius, Murray Wham, Bryan Whelan, Nicole White, Aurélie Wiedemann, Surya Otto Wijaya, Virginie Williams, Patricia J. Williams, Bailey Williams, Evert-Jan Wils, Karolina Witt, Jessica Wittman, Xin Ci Wong, Yew Sing Wong, Teck Fung Wong, Calvin Wong, Lim Saio Xian, Ioannis Xynogalas, Siti Rohani Binti Mohd Yakop, Masaki Yamazaki, Elizabeth Yarad, Yazdan Yazdanpanah, Nicholas Yee- Liang Hing, Abdelrahman Yehia Mahmoud Abdelaal, Cécile Yelnik, Chian Hui Yeoh, Touxiong Yiaye, Hodane Yonis, Obada Yousif, Saptadi Yuliarto, Akram Zaaqoq, Marion Zabbe, Masliza Zahid, Nor Zaila Binti Zaidan, Maria Zambon, Miguel Zambrano, Alberto Zanella, Konrad Zawadka, Nurul Zaynah, Hiba Zayyad, Alexander Zoufaly, David Zucman

**Affiliations:** 1 Intensive Care Unit 4, Department of Intensive Care, São Francisco Xavier Hospital, ULSLO, Lisbon, Portugal; 2 Nova Medical School, Clinical Medicine, CHRC, NOVA University of Lisbon, Lisbon, Portugal; 3 Department of Intensive Care Hopital, Universitaire de Bruxelles (HUB), Université Libre de Bruxelles (ULB), Brussels, Belgium; 4 Unisabana Center for Translational Science, School of Medicine, Chia, Colombia; 5 ISARIC, Pandemic Sciences Institute, University of Oxford, Oxford, UK; 6 Université de Lorraine, CHRU-Nancy, Service des Maladies Infectieuses et Tropicales, F-54000, Nancy, France; 7 Department of Anesthesiology and Intensive Therapy RSUD Dr Saiful Anwar Jawa Timur, Brawijaya University Malang East Java, Malang, Indonesia; 8 Center for Clinical Epidemiology and Research Unit of Clinical Epidemiology, OUH Odense University Hospital, 5000 Odense C, Denmark; 9 Université de Lorraine, Inserm, INSPIIRE, F-54000, Nancy, France

**Keywords:** Lymphopenia, Immunity, Sepsis, In-hospital mortality, COVID-19, Multistate analysis, Corticosteroids

## Abstract

**Objective:**

Immune dysregulation plays a pivotal role in the pathophysiology of sepsis and COVID-19, with lymphopenia emerging as a consistent marker of severity and poor prognosis. However, most existing studies have assessed lymphocyte counts at isolated time points, limiting insights into their temporal behavior and prognostic value. The dynamics of lymphocyte recovery or persistence of lymphopenia remain largely unexplored in large populations, as well as the impact of adjunctive therapies such as corticosteroids. We hypothesized that the persistence or recovery of lymphopenia may be key to understanding disease progression and predicting outcomes. Using the multinational ISARIC cohort, we investigated longitudinal lymphocyte trajectories in hospitalized patients and the clinical determinants associated with their evolution over time.

**Methods:**

We conducted a multinational prospective observational cohort study using data from the ISARIC-WHO Clinical Characterization Protocol. Patients with confirmed SARS-CoV-2 infection and at least four lymphocyte measurements during the first 28 days of hospitalization were included. We analyzed lymphocyte trajectories, Cox regression survival analyses and multivariable linear regression modelling. We also applied multistate models and joint modeling to assess the association between lymphocyte trajectories and 28-day mortality, incorporating corticosteroid use as a time-varying covariate.

**Results:**

Of 945,317 screened patients, 231,933 hospitalized adults with confirmed COVID-19 and sufficient lymphocyte data were included, with 56.6% classified as lymphopenic. Lymphopenia was independently associated with higher rates of ICU admission, organ support, and in-hospital mortality (OR = 1.52, 95% CI 1.48–1.55), and lower absolute lymphocyte counts were strongly linked to worse survival in adjusted Cox models (HR = 1.33 per 1 × 10⁹ cells/L decrease, 95% CI 1.28–1.38). Multistate modeling revealed that lymphopenic patients had a significantly higher daily transition rate to death and a shorter duration in that immune state, while corticosteroid exposure was associated with an increased likelihood of entering and remaining in lymphopenia. Joint modeling identified age, sex, and corticosteroid use as significant predictors of lower lymphocyte trajectories over time, with distinct dynamics between survivors and non-survivors.

**Conclusion:**

Lymphopenia was common and strongly associated with worse outcomes in hospitalized COVID-19 patients, with impaired recovery particularly evident in those receiving corticosteroids. These findings highlight the value of lymphocyte monitoring to inform tailored immunomodulatory strategies in sepsis and severe viral infections.

**Supplementary Information:**

The online version contains supplementary material available at 10.1186/s40635-026-00864-x.

## Introduction

Immune dysfunction has gained prominence as a key area of interest in the pathophysiology of sepsis, with compelling evidence linking the profound derangement in the host inflammatory response to clinical outcomes [1, 2]. Our long-standing view of a universal immune activation response towards infection has been fundamentally redefined. Recent evidence clearly suggests a far more dynamic and heterogeneous biological mechanism, with septic patients transitioning from early hyperinflammation and cytokine release to a state of immune paralysis, usually persisting or fluctuating, in and out of an immunosuppressive phase [3–7]. This complexity is potentially correlated with a wide variability in outcomes among septic patients, prompting increasing interest in immunophenotypes and distinct immune response patterns, despite similar infectious insults [8, 9]. The COVID-19 pandemic intensified focus on this paradigm, revealing striking interindividual differences in disease trajectories despite a common viral insult. This variability has exposed the limitations of our current understanding of immune dynamics over time.

Among the various immunological alterations observed in COVID-19 and other causes of sepsis, lymphopenia has consistently emerged as a unique biological marker of disease severity and poor prognosis [10]. Absolute lymphocyte counts below 1 × 10^9^ cells/L have been repeatedly associated with increased risk of organ failure, need for organ support, and mortality. However, most existing studies have relied on single time-point measurements, limiting our understanding of the dynamics of lymphocyte recovery or persistence of immune suppression [11–15]. A recent meta-analysis by Elçioğlu et al*.* demonstrated consistently higher in-hospital mortality rates, both early and late, across fifteen observational studies, arguing that longitudinal immune trajectories may provide greater insight into clinical outcomes [16]. Yet, whether lymphopenia reflects a transient epiphenomenon, a marker of adaptive exhaustion, or a surrogate of immune failure remains unclear, particularly when evaluated outside of its temporal context [17].

Despite consistent associations between lymphopenia and adverse outcomes in critical illness, the temporal dimension of lymphocyte trajectories remains poorly understood. As such, the prognostic implications of lymphocyte dynamics, whether as markers of immune recovery or ongoing dysfunction, remain largely unexplored. In this context, we aimed to assess the prognostic value of lymphocyte trajectories over time. Leveraging the International Severe Acute Respiratory and Emerging Infection Consortium (ISARIC) COVID-19 database, one of the largest standardized, multinational cohorts of hospitalized patients, we conducted a comprehensive analysis of hospitalized COVID-19 patients with serial lymphocyte counts. We aimed to describe the presence of lymphopenia in these patients and assess its persistence, resolution, or fluctuation over time, exploring how dynamic patterns relate to clinical outcomes, as well as the impact of adjunctive therapies such as corticosteroids.

## Methods

This study is a prospective observational cohort study of hospitalized patients from five continents. The study Consortium framework is provided by the International Severe Acute Respiratory and Emerging Infection (ISARIC)—World Health Organization (WHO) Clinical Characterization Protocol for Severe Emerging Infections [18, 19]. The protocol, case report forms, consent forms, and study information are available on the ISARIC website (https://isaric.org). This standardized protocol uses tiered data collection tailored to a range of resource settings. Investigators from 69 countries collected prospective data using the ISARIC case report form (CRF) built on Research Electronic Data Capture (REDCap, version 8.11.11, Vanderbilt University, Nashville, Tenn.) [20] hosted by the University of Oxford. Other investigators collected data using locally hosted systems and submitted it to the ISARIC dataset for centralized mapping. All investigators retain full rights to their data.

This observational study required no change to clinical management and encouraged patient enrolment in other research projects. The ISARIC-WHO Clinical Characterization Protocol was approved by the World Health Organization Ethics Review Committee (RPC571 and RPC572). Also, local ethics approval was obtained for each participating country and site according to local requirements.

### Study population

We included all adult patients hospitalized with confirmed SARS-CoV-2 infection, defined by clinical and radiological criteria (i.e., symptoms and findings of SARS-CoV-2 pneumonia seen in thoracic diagnostic images) and/or a positive reverse transcription-polymerase chain reaction (RT-PCR) result from a respiratory sample. Eligible patients had to be biochemically characterized during hospitalization, with available data on absolute lymphocyte and leukocyte counts. Patients with > 30% missing data in variables deemed critical for analysis were excluded (see the Electronic Supplementary Material [ESM] for further details).

To ensure a meaningful assessment of lymphocyte dynamics, we excluded patients who lacked data for at least four absolute lymphocyte count measurements during the first 28 days of hospitalization. These measurements were required to include values from days 0, 1, 3, and 7, allowing consistent evaluation of early immune trajectories during the critical initial phase of the septic process and ensuring that lymphocyte counts were obtained at standardized time points across all patients. Patients with implausible values for these variables or with missing outcome data were also excluded. Censoring due to early discharge or death was handled by including all available data up to the time of censoring, ensuring that incomplete follow-up did not bias the estimation of early lymphocyte dynamics.

### Data collection and definitions

Demographic, clinical, and biochemical data were extracted from the electronic case report forms (eCRFs), including age, sex, comorbidities, country income classification (per World Bank definitions: https://data.worldbank.org/country), vital signs at admission, laboratory values during hospitalization, need for advanced organ support (mechanical ventilation, vasopressors, renal replacement therapy), systemic complications, and treatments administered during hospitalization. All study variables were predefined in the ISARIC study protocol and case report form completion guide is available online (https://isaric.org).

The resulting analytic cohort is referred to as the Epidemiological cohort. Patients were further stratified based on the development of lymphopenia, defined as at least one absolute lymphocyte count < 1 × 10⁹ cells/L during hospitalization [10, 21]. Patients with no such values were classified in the non-lymphopenia group. For all analyses, day 0 was considered the day of hospital admission.

### Outcomes

The primary outcome evaluated in this study was 28-day all cause in-hospital mortality. Secondary outcomes included the rate and risk factors for Lymphopenia development, rate of advanced organ support requirements during the hospitalization episode (i.e. mechanical ventilation, vasopressor support or renal replacement therapy), the rate of ICU admission, the ICU and hospital all-cause mortalities and the impact of corticosteroids on lymphocyte trajectories and remaining outcomes.

### Statistical methods

Data was expressed as mean (standard deviation [SD]), if normally distributed, and non-normally distributed variables as median (interquartile range [IQR]). Categorical variables were expressed as numbers and percentages. Chi-square test was used for categorical variables, and t-test and Kruskal–Wallis were used on continuous variables for statistical assessment of outcomes between groups. Initially, we performed univariate analysis of demographic, comorbidities, clinical presentation, biochemical characterization, complications and outcomes between Lymphopenia and non-Lymphopenia cohorts. Absolute lymphocyte counts distribution over time was also obtained to compare the cohorts.

Survival analysis was performed using Kaplan–Meier curves with log-rank tests to compare 28-day survival across lymphocyte-defined groups. Multivariable Cox proportional hazards models evaluated the association between continuous lymphocyte counts and mortality, adjusting for age, sex, corticosteroid treatment, and organ support interventions (vasopressors/inotropes, renal replacement therapy, invasive and non-invasive ventilation). Proportional hazards assumptions were verified.

Additionally, we conducted a multivariable logistic regression to assess associations between lymphopenia status (based on longitudinal lymphocyte thresholds) and all-cause in-hospital mortality, adjusting for the same covariates. Model discrimination and calibration were evaluated.

To analyze transitions between the non-lymphopenic and lymphopenic states and the risk of death, we fitted a continuous-time, time-homogeneous multistate Markov model using Aalen-Johansen estimation. This approach allowed estimation of transition probabilities over time, capturing competing risks and intermediate transitions, and was adjusted for age and gender. An additional analysis including corticosteroid therapy as a covariate was performed subsequently. A diagram of the multistate analysis is presented in the Methods section of the ESM.

To jointly assess lymphocyte dynamics and mortality in relation to corticosteroid treatment, a joint modeling approach combined a linear mixed-effects model for longitudinal lymphocyte counts with a Cox survival model. Corticosteroid treatment was included as a time-varying covariate. Models were adjusted for relevant clinical variables, organ support requirements (respiratory, hemodynamic and renal) and demographic factors.

Statistical analyses were performed using R version 4.4.0 (R Foundation for Statistical Computing, Vienna, Austria) with packages *survival, lme4* and *JMbayes2*. A two-sided p-value < 0.05 was considered statistically significant.

## Results

**Fig. 1:**
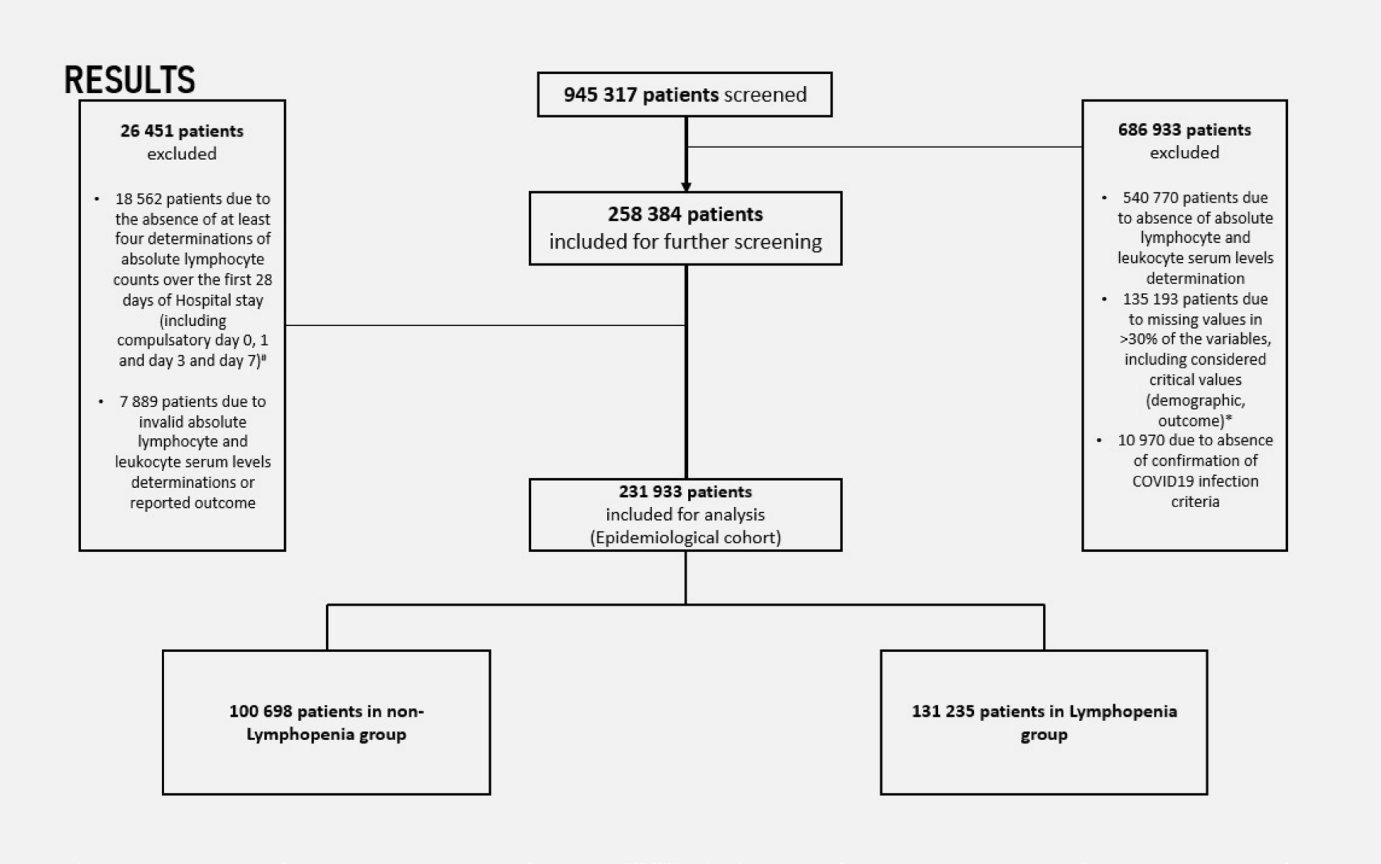
Patient selection Diagram using STROBE requirements. * Considered critical variables are further characterized in the ESM; # Day 0 was defined as the day of hospital admission. Required days D0, D1, D3 and D7 correspond to days after hospital admission

A total of 945,317 patients were initially screened for inclusion. Among these, 686,933 were excluded due to the absence of valid absolute lymphocyte and leukocyte determinations, high rate of missing values or absence of confirmation of required COVID-19 infection criteria. After applying exclusion criteria, 26,451 patients were removed, resulting in a final cohort of 231,933 patients (as depicted in Fig. 1). Of these, 100,698 patients (43.4%) were classified into the non-Lymphopenia group, while 131,235 patients (56.6%) were categorized in the Lymphopenia group.

### Patients’ demographic, treatment and outcome characteristics

**Fig. 2:**
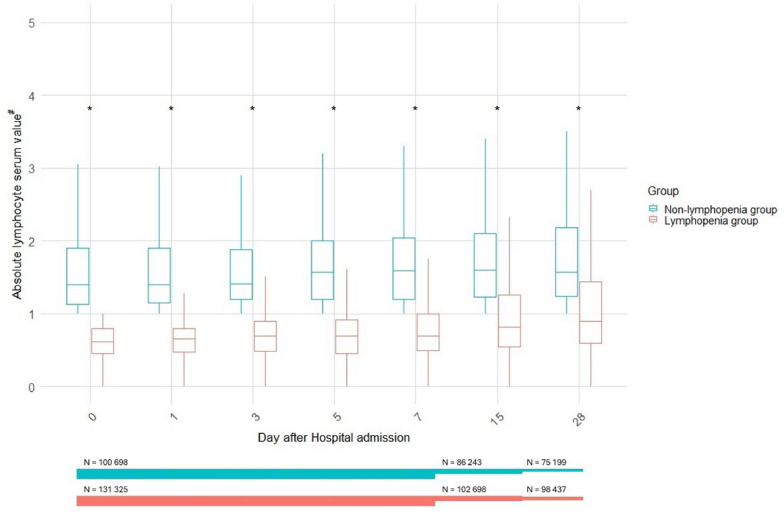
Comparison of absolute lymphocyte count between patients in the Lymphopenia and non-Lymphopenia groups. ^#^Values are expressed in × 10^9^ cells/L

Patient characteristics are detailed in Table 1. The groups presented significantly different demographic and clinical characteristics, with Lymphopenia group being composed of older patients, with a male preponderance and consistently higher rates of comorbidities and complications during Hospital stay. Patients in the Lymphopenia group also required higher rates of ICU admission and longer lengths of respiratory support (invasive mechanical ventilation) along with longer lengths of ICU and Hospital stay. We also documented higher all-cause mortality rates, either at ICU or in-Hospital, in the Lymphopenia group when compared to non-Lymphopenia group patients (Table 1). Figure 2 illustrates the longitudinal distribution of absolute lymphocyte serum values across Day 0 to Day 28 stratified by lymphopenia status. Patients classified in the Lymphopenia group consistently exhibited significantly lower lymphocyte counts at every measured time point compared to the non-Lymphopenia group (*p* < 0.05 for all days). This persistent lymphopenia was evident despite the general upward trend in lymphocyte values observed over time in both groups.

**Table 1:** Demographic, clinical, treatment and outcome characteristics stratified by lymphopenia groups

Variable	Non-Lymphopenia (n = 100,698)	Lymphopenia (n = 131,235)	p-value
Demographic Variables			
Age, years (median [IQR])	60.0 [43.0, 76.0]	69.0 [55.0, 80.0]	** < 0.001**
Gender, males (%)	51,834 (51.5)	79,285 (60.4)	** < 0.001**
Height (cm), Median [IQR]	168.0 [161.0, 175.0]	170.0 [163.0, 175.0]	** < 0.001**
Weight (kg), Median [IQR]	75.0 [54.0, 90.0]	76.0 [60.0, 90.0]	0.410
Comorbidities			** < 0.001**
HIV/AIDS	653 (0.8%)	562 (0.5%)	
Asthma	12,102 (13.6%)	15,338 (13.0%)	
Chronic Cardiac Disease	19,958 (21.9%)	33,659 (27.7%)	
Chronic Hematological Disease	2,782 (3.1%)	5,216 (4.5%)	
Chronic Kidney Disease	9,905 (11.1%)	18,518 (15.5%)	
Chronic Neurological Disorder	8,592 (9.6%)	11,610 (9.9%)	
Chronic Pulmonary Disease	11,301 (12.4%)	19,704 (16.3%)	
Dementia	7,884 (8.8%)	11,635 (9.8%)	
Diabetes	23,275 (25.8%)	31,204 (26.1%)	
Hypertension	31,899 (39.0%)	48,248 (46.1%)	
Immunosuppression	1,272 (3.1%)	3,069 (5.1%)	
Liver Disease	2,768 (3.0%)	3,897 (3.2%)	
Malignant Neoplasm	6,119 (6.8%)	12,958 (10.8%)	
Malnutrition	1,426 (1.7%)	2,089 (1.9%)	
Obesity	13,738 (16.8%)	17,678 (16.5%)	
Other Comorbidities	35,202 (45.4%)	50,732 (48.9%)	
Rare Diseases/Inborn Errors of Metabolism	295 (0.7%)	320 (0.5%)	
Rheumatologic Disorder	8,254 (9.3%)	12,635 (10.8%)	
Smoking	22,382 (38.9%)	32,512 (43.6%)	
Transplantation	468 (1.1%)	1,217 (2.0%)	
Tuberculosis	46 (0.5%)	71 (0.5%)	
Pregnancy	2,471 (12.7%)	1,648 (11.0%)	
Symptoms at hospital admisssion			** < 0.001**
Abdominal Pain (%)	7822 (10.0)	9510 (9.3)	
Altered Consciousness/Confusion (%)	13,259 (17.7)	22,477 (21.2)	
Asymptomatic (%)	2386 (4.6)	1973 (2.7)	
Bleeding (%)	1407 (1.8)	1735 (1.7)	
Chest Pain (%)	13,443 (17.0)	14,960 (14.4)	
Conjunctivitis (%)	327 (0.4)	374 (0.4)	
Cough (%)	49,945 (58.7)	76,617 (67.1)	
Diarrhoea (%)	12,996 (16.2)	19,947 (18.8)	
Ear Pain (%)	305 (0.5)	312 (0.4)	
Fatigue/Malaise (%)	29,943 (38.7)	46,663 (45.7)	
Headache (%)	9787 (13.1)	11,586 (11.8)	
History of Fever (%)	44,881 (53.2)	73,516 (64.8)	
Lost/Altered Sense of Smell (%)	5255 (8.1)	6014 (7.5)	
Lost/Altered Sense of Taste (%)	5801 (9.2)	7420 (9.5)	
Lymphadenopathy (%)	366 (0.5)	486 (0.5)	
Muscle Aches/Joint Pain (%)	13,836 (18.6)	19,434 (19.8)	
Runny Nose (%)	2710 (3.8)	2606 (2.8)	
Seizures (%)	1038 (1.4)	1084 (1.1)	
Severe Dehydration (%)	4308 (11.1)	7032 (13.9)	
Shortness of Breath (%)	50,301 (58.9)	81,392 (70.6)	
Skin Rash (%)	1639 (2.2)	2362 (2.4)	
Sore Throat (%)	5747 (7.9)	6506 (6.9)	
Vomiting/Nausea (%)	15,177 (19.0)	20,961 (19.9)	
Wheezing (%)	4550 (6.0)	7141 (7.3)	
Complications during hospital stay			** < 0.001**
Acute Kidney Injury (%)	10,223 (12.4)	20,689 (18.4)	
Anemia (%)	8833 (10.7)	18,178 (16.3)	
ARDS (%)	7481 (9.1)	18,587 (16.8)	
Bacteremia (%)	2370 (2.9)	6134 (5.5)	
Bacterial Pneumonia (%)	8590 (10.6)	17,623 (16.2)	
Bleeding (%)	17 (0.8)	13 (0.9)	
Cardiac Arrest (%)	1549 (1.9)	3731 (3.4)	
Cardiac Arrhythmia (%)	4450 (5.6)	8883 (8.4)	
Cardiac Ischemia (%)	901 (1.1)	1690 (1.5)	
Cardiomyopathy (%)	273 (0.4)	593 (0.6)	
Coagulation Disorder (%)	2673 (3.3)	5628 (5.1)	
Congestive Heart Failure (%)	1818 (2.2)	3629 (3.3)	
Cryptogenic Organizing Pneumonia (%)	250 (0.3)	644 (0.6)	
Deep Vein Thrombosis (%)	281 (0.7)	445 (0.8)	
DIC (%)	2 (0.8)	0 (0.0)	
Endocarditis (%)	48 (0.4)	86 (0.4)	
GI Bleeding (%)	919 (1.1)	1538 (1.4)	
Hemodynamic Decompensation (%)	159 (8.1)	178 (12.8)	
Hyperglycemia (%)	8259 (10.1)	15,559 (14.1)	
Liver Dysfunction (%)	4902 (6.0)	9333 (8.4)	
Meningitis/Encephalitis (%)	172 (0.2)	249 (0.2)	
Myocardial Infarction (%)	98 (0.9)	217 (1.2)	
Neurological Complication (%)	1351 (2.5)	2136 (2.8)	
Other Complications (%)	17,013 (23.1)	27,324 (26.8)	
Pleural Effusion (%)	3227 (3.9)	6809 (6.1)	
Pneumothorax (%)	754 (0.9)	2121 (1.9)	
Pulmonary Embolism (%)	1736 (3.5)	2929 (4.1)	
Pulmonary Embolism/DVT (%)	34 (12.5)	86 (15.0)	
Rhabdomyolysis (%)	341 (0.4)	762 (0.7)	
Seizures (%)	850 (1.0)	961 (0.9)	
Stroke (%)	1130 (1.4)	1460 (1.3)	
Supraventricular Arrhythmia (%)	45 (2.2)	61 (4.3)	
Thromboembolism (%)	9 (0.8)	38 (1.9)	
Thrombosis (%)	84 (4.1)	109 (7.7)	
VAP (%)	99 (36.4)	264 (46.2)	
Viral Pneumonia (%)	38,493 (47.1)	66,165 (59.3)	
Main outcomes			
ICU Admission	17,183 (17.6%)	35,281 (27.7%)	** < 0.001**
Duration of IMV, days (median [IQR])	6.0 [3.0, 12.0]	7.0 [4.0, 14.0]	** < 0.001**
Duration of Prone Ventilation, days (median [IQR])	4.0 [2.0, 8.0]	4.0 [2.0, 7.0]	0.055
Duration of HFNC, days (median [IQR])	4.0 [2.0, 7.0]	5.0 [3.0, 7.0]	0.226
Duration of ECMO, days (median [IQR])	9.0 [3.0, 11.5]	8.0 [4.0, 13.8]	0.756
Duration from Admission to ICU, days (median [IQR])	1.0 [0.0, 3.0]	1.0 [0.0, 3.0]	** < 0.001**
Duration from Admission to IMV, days (median [IQR])	1.0 [0.0, 5.0]	2.0 [0.0, 5.0]	** < 0.001**
Duration of ICU (median [IQR])	7.0 [3.0, 13.0]	9.0 [4.0, 17.0]	** < 0.001**
Duration of Hospital Stay (median [IQR])	7.0 [3.0, 13.0]	9.0 [4.0, 16.0]	** < 0.001**
In-Hospital outcome			
Death	12,654 (12.6)	32,653 (24.9)	** < 0.001**
Discharge	74,361 (73.9)	79,969 (61.0)	** < 0.001**
Ongoing Care	1,593 (1.6)	2,968 (2.3)	** < 0.001**
Transferred	4,975 (4.9)	6,684 (5.1)	0.124

Data is median [IQR] or n (%). *DIC* Disseminated intravascular coagulation disorder, *GI* Gastrointestinal, *VAP* Ventilator-acquired pneumonia, *ICU* intensive care unit, *IMV* Invasive mechanical ventilation, *HFNC* High-flow nasal cannula, *ECMO* extracorporal membrane oxygenation

### Primary outcomes and survival analysis

**Fig. 3:**
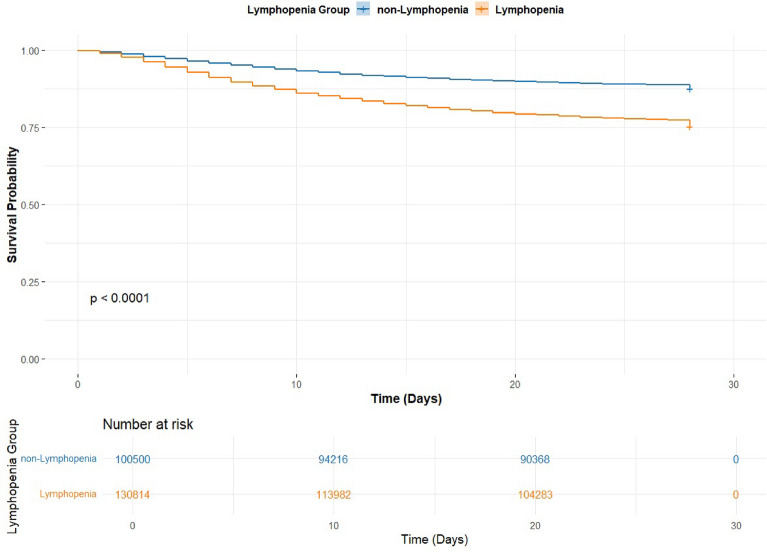
Kaplan–Meier survival curves and analysis of cumulative survival, including log-rank test, between Lymphopenia and non-Lymphopenia groups

The adjusted multivariable Cox proportional hazards regression demonstrated that lower lymphocyte counts were independently associated with increased risk of death (HR = 1.33 per 1 × 10^9^ cells/L, 95% CI 1.28–1.38, *p* < 0.001). Increasing age (HR = 1.05 per year, 95% CI 1.05–1.06, *p* < 0.001), male sex (HR = 1.22, 95% CI 1.20–1.24, *p* < 0.001), corticosteroid use (HR = 1.05, 95% CI 1.03–1.07, *p* < 0.001) and vasopressor, mechanical ventilation and renal replacement therapy requirements (all p < 0.001) were also significantly associated with increased hazard of death. A forest plot of hazard ratios is presented in Fig. 1 ESM. These results were consistent with the survival estimates by lymphocyte status, illustrated with a Kaplan–Meier curve in Fig. 3, with differences in survival confirmed by the log-rank test (*p* < 0.001).

We also performed a multivariable logistic regression to identify independent predictors associated with the all-cause in-hospital mortality (Table 2). After adjustment for key confounders, lymphopenia status was independently associated with a significantly increased odds of the adverse outcome (OR 1.52, 95% CI 1.48–1.55, *p* < 0.001). Other significant predictors included advancing age (OR 1.07 per year increase, 95% CI 1.07–1.07, *p* < 0.001) and male sex (OR 1.29, 95% CI 1.26–1.32, *p* < 0.001). Use of corticosteroids (OR 1.20, 95% CI 1.17–1.23, *p* < 0.001) and organ support requirements were also linked to higher odds of the outcome.

**Table 2:** Multivariable logistic regression predicting all-cause in-hospital mortality

Variable	Odds Ratio (OR)	95% Confidence Interval	*p*-value
Lymphopenia group (yes)	1.52	1.48–1.55	< 0.001
Age (years, continuous)	1.07	1.03–1.12	< 0.001
Gender (Male)	1.29	1.26–1.32	< 0.001
Corticosteroid treatment (yes)	1.20	1.17–1.23	< 0.001
Inotropes/Vasopressors requirement (yes)	1.39	1.31–1.47	< 0.001
Renal replacement therapy requirement (yes)	2.55	2.38–2.73	< 0.001
Invasive mechanical ventilation requirement (yes)	3.51	3.33–3.70	< 0.001
Non-invasive ventilation requirement (yes)	1.91	1.84–1.99	< 0.001

**Fig. 4:**
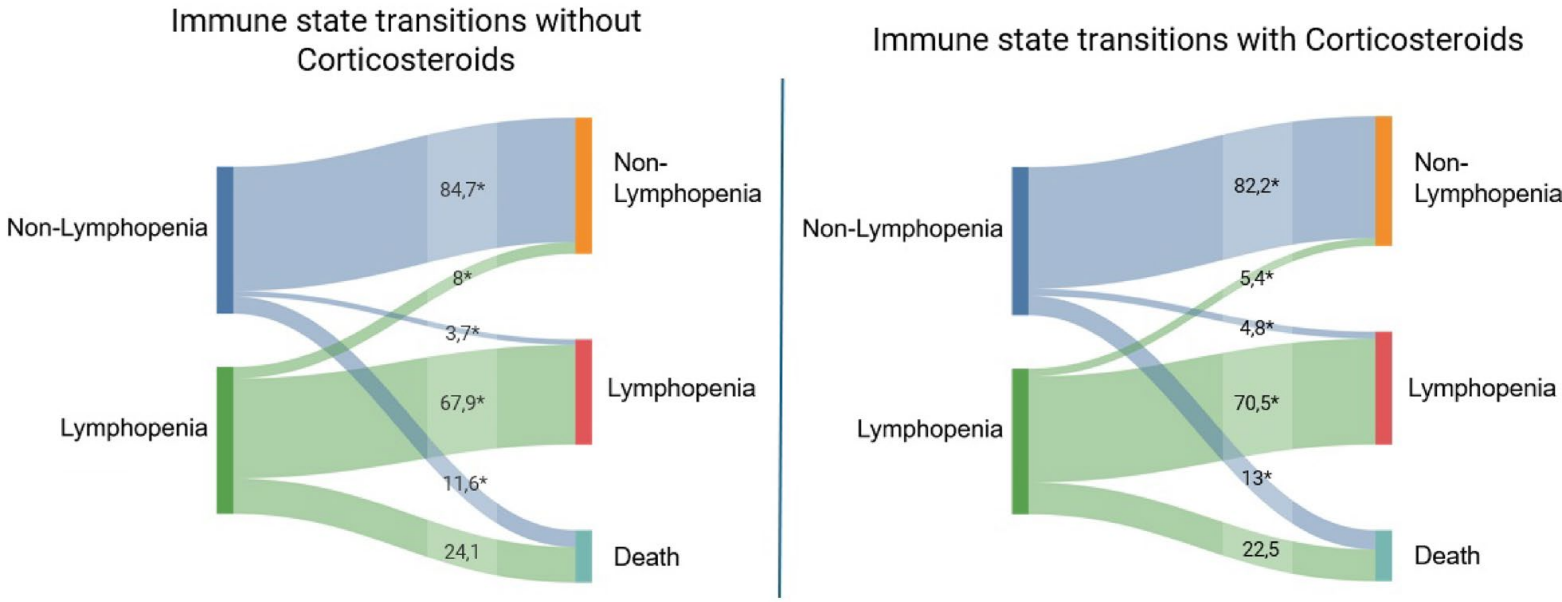
Modified alluvial plot depicting transition rates of Lymphocyte status and mortality outcomes overtime, in patients untreated and treated with corticosteroids. The figure illustrates transitions between three clinical states—lymphopenia, non-lymphopenia, and death. Each flow represents a dynamic variable assessed longitudinally, defined by the percentage of patients moving from one state to another in accordance with the group of origin, with the width of the stream proportional to that percentage of individuals. Transitions culminating in death are shown as terminal flows representing an absorbing state. * represents a significant difference between groups for the referred transition

Estimated transition intensities between states of our multistate Markov model are depicted in Table 3. Patients in the non-Lymphopenia state transitioned to the Lymphopenia state at a rate of 1.2% per day (95% CI 1.18–1.23) and to death at a rate of 2.0% per day (95% CI 1.99–2.07). From the Lymphopenia state, the transition intensity back to the non-Lymphopenia state was 1.6% per day (95% CI 1.54–1.61), while the transition to death occurred at a markedly higher rate of 4.3% per day (95% CI 4.26–4.36). All transitions were statistically significant (p < 0.001). Mean expected duration spent in a state before transitioning were 30.9 days for the non-Lymphopenia state and 17.1 days for the Lymphopenia state. These estimates suggest differing stability across immune states, with shorter durations observed in lymphopenia patients. In a covariate-adjusted multistate model including corticosteroid exposure, corticosteroid use was associated with increased hazard of transitioning from the non-Lymphopenia state to Lymphopenia (HR = 1.13, 95% CI 1.08–1.18) and from non-Lymphopenia to death (HR = 1.16, 95% CI 1.12–1.20). Conversely, corticosteroid use was associated with a reduced hazard of transitioning from the Lymphopenia to the non-Lymphopenia state (HR = 0.71, 95% CI 0.68–0.74), with no significant effect on transitions from Lymphopenia to death (HR = 0.94, 95% CI 0.92–1.06) (Fig. 4, Fig. 2 ESM).

**Table 3:** Estimated transition intensities between immune states per day

From → To	Non-lymphopenic	Lymphopenic	Death
Non-lymphopenic	–	1.205 (1.177–1.233)	2.030 (1.995–2.066)
Lymphopenic	1.574 (1.544–1.605)	–	4.311 (4.262–4.362)
Death (absorbing state)	–	–	–

Values represent estimated transition intensities (hazard rates per day) with 95% confidence intervals. They reflect the relative speed of transitions rather than absolute transition probabilities

### Longitudinal absolute lymphocyte serum count trajectories and impact of corticosteroids

**Fig. 5:**
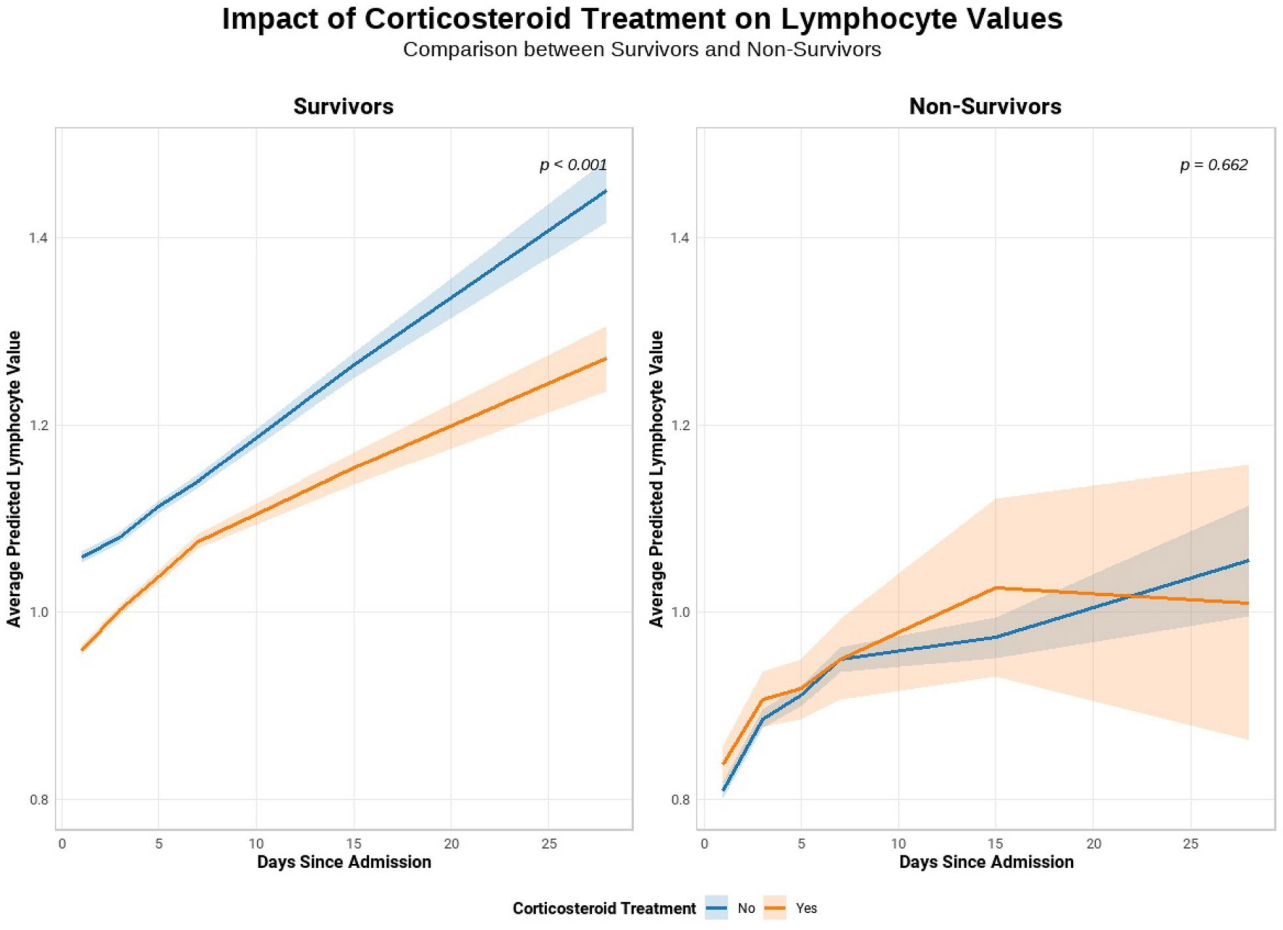
Joint model combining a linear mixed-effects model and Cox regression, depicting the impact of corticosteroid therapy on predicted absolute lymphocyte counts (× 10⁹ cells/L) over time, in surviving and non-surviving patients

Using a joint modeling approach, our data revealed multiple significant predictors of lymphocyte trajectories. Increasing age and male sex were associated with lower lymphocyte counts, corresponding to relative decreases of approximately 13% per scaled year of age (95% CI: 11% to 15%) and 11% lower counts in males compared to females (9% to 14% decrease; both p < 0.001). Corticosteroid treatment was linked to a significant 10% reduction in lymphocyte counts over time (95% CI: 7% to 13% decrease, p < 0.001). Additional factors such as inotropes/vasopressors (approximately 10% decrease), renal replacement therapy (approximately 12% increase), and non-invasive ventilation (approximately 15% decrease) also showed significant effects on lymphocyte trajectories. Among survivors, lymphocyte values were significantly influenced by time since admission, corticosteroid treatment, and their interaction (p < 0.005 for all), indicating that corticosteroids modulate lymphocyte dynamics over time in this group. In non-survivors, corticosteroid treatment and its interaction with time were not significant, though lymphocyte counts still changed significantly over time (p = 0.0007). Overall, survivors and non-survivors differed significantly in lymphocyte counts, with the interaction between time and outcome being more pronounced in patients not receiving corticosteroids (Fig. 5).

## Discussion

Our findings robustly position lymphopenia as a prevalent and clinically meaningful marker of immune dysregulation in hospitalized patients with COVID-19. This study also documents that the presence and persistence of lymphopenia were independently associated with a markedly increased risk of adverse outcomes, including ICU admission, requirement of organ support, and higher in-hospital mortality. It also supports that patients who developed progressive or sustained lymphopenia, largely regardless of their initial lymphocyte count at admission, faced significantly higher mortality risk. These results suggest lymphopenia not merely as an epiphenomenon of critical illness but as a dynamic, prognostically relevant immunological signature.

Lymphopenia has long been proposed as a marker of immune dysregulation in sepsis and severe viral infections, despite the absence of definitive large-scale evidence support [22]. Our findings confirm and extend prior observations by providing multivariable-adjusted analyses that consistently link lower lymphocyte counts with increased hazard of in-hospital complications (across nearly every organ system), ICU admission rates, organ support requirements, and death. Although with a pragmatic and far less restrictive definition of lymphopenia status, demanding a single measurement during the first 28 days of hospital stay, our results show that over half of COVID-19 hospitalized patients (56%) met criteria for lymphopenia, exceeding prevalence estimates from earlier studies [15, 23, 24].

Patients who exhibit lymphopenia had significantly higher risk of adverse outcomes, even after adjustment for major clinical and treatment-related determinants, with 52% higher odds of in-hospital death compared to non-lymphopenia patients. These findings are consistent with previous studies, suggesting a potential dose–response relationship between lymphocyte depletion and poor prognosis [16, 25, 26]. These data support the inclusion of immune monitoring, specifically lymphocyte kinetics, as a critical parameter in the clinical assessment of hospitalized patients, comparable to the monitoring of other organ systems.

Our multistate model further reveals the temporal instability of immune states, showing that patients transitioning into lymphopenia face a markedly higher daily risk of death, while recovery from this state, or its complete absence, is associated with a survival advantage. These findings offer compelling support for the hypothesis that restoration of immune competence, supported by lymphocyte recovery, may represent a potentially critical inflection point in the trajectory of these hospitalized patients. Consistent with prior evidence, this dynamic is likely not limited to COVID-19, but may extend to other etiologies of sepsis, given the well-documented role of immune recovery in various infectious contexts [27–29].

Our data also reinforces the paradoxical role of corticosteroids, which were independently associated with both increased risk of transitioning into lymphopenia, impaired lymphocyte recovery and higher mortality. Considering their immunosuppressive properties, our results may reflect a potential unintended cost on immune resilience, particularly in patients with already compromised adaptive responses [29, 30]. Notably, in survivors, corticosteroids appeared to exert dynamic modulatory effects on lymphocyte trajectories. In contrast, in non-survivors, these modulatory effects appeared blunted, suggesting differential responsiveness based on host immune trajectory and possibly timing of administration. These results are consistent with recent data from several studies challenging the “one-size-fits-all” approach to corticosteroid therapy, specifically in COVID-19, and more broadly in sepsis [31–33]. A recent meta-analysis argues that the observed survival benefit of corticosteroids in COVID-19 appeared to be largely driven by the RECOVERY trial, as the effect was no longer statistically significant when this study was excluded [34, 35]. Another meta-analysis by Xiangrong Ye et al. demonstrated no significant difference in 28-day mortality among hospitalized COVID-19 patients receiving corticosteroids [36]. However, subgroup analyses suggested potential clinical benefit in moderate-to-severe cases, particularly in selected populations, thereby advocating for a personalized therapeutic approach aimed at minimizing adverse immunological consequences. Importantly, the absence of data on steroid treatment and lymphocyte trajectories in those analyses may limit the interpretation of these findings even on selected populations.

Collectively, all this data contributes to the growing body of evidence framing immune paralysis and dysfunction as a clinically relevant, and often underutilized, readily accessible surrogate for immune trajectory. This is particularly important in contexts where advanced immunophenotyping is unavailable [37]. Cautiously, our results also argue against a reductionist interpretation: lymphocyte counts alone cannot fully characterize the complexity of host immune response in sepsis and severe viral infections. Nevertheless, lymphocyte trajectories may serve as valuable biomarkers when interpreted within a broader immunological context, including patient age, comorbidities, disease phase, and therapeutic exposures, namely corticosteroids.

Our study benefits from several strengths. It is based on a large sample size, with granular temporal resolution of both laboratory and clinical variables. The application of advanced statistical methodologies, including multistate modeling and latent class mixed models to capture dynamic immunological transitions and associations between lymphocyte dynamics, treatment exposures, and outcomes. Furthermore, it uses a cohort with pragmatic data capturing across multiple countries that represents real-world reported observations.

However, some limitations merit consideration. First, the observational nature of the study precludes definitive causal inferences, and residual confounding cannot be fully excluded. Second, missing laboratory data led to the exclusion of a substantial number of patients, possibly limiting the power and generalizability of some of our more sophisticated analyses, such as LCMM. Also, most patients in this cohort were from high-income countries, which may further limit generalizability. Additionally, we cannot exclude that lymphocyte measurements may have been performed more frequently in patients with severe disease, which could introduce selection bias; however, our inclusion criteria requiring measurements on days 0, 1, 3, and 7 were applied consistently across all patients, in attempt to reduce this impact, although not fully eliminating this potential bias. To ensure the integrity of the dataset, we opted not to impute missing values in the primary analyses; sensitivity analyses using median imputation and last observation carried forward confirmed that our main findings were robust and did not materially change. We also acknowledge that requiring at least four lymphocyte measurements within the first week of hospitalization, to reliably characterize longitudinal trajectories and immune dysregulation, may have excluded patients who died, were discharged, or transferred early, introducing potential survivorship bias. Nevertheless, including patients with only one or two measurements would have resulted in misclassification of lymphocyte status and unreliable modeling of dynamic changes, which could have biased the study in other ways. This trade-off was an intentional methodological choice to ensure robustness of trajectory-based analyses. Third, we agree that the definition of lymphopenia employed, while intentionally inclusive, may lack sensitivity and specificity. We aimed to capture all plausible immune states consistent with lymphopenia in every patient. Additionally, we state that lymphocyte count is a crude proxy for immune function, and more refined markers (e.g., CD4 + T cells absolute count, mHLA-DR expression, PD-1 and PD-L1 serum levels or Ex-vivo LPS-induced tests) were not available. Also of note, the lack of granular data on corticosteroid type, cumulative dose, and duration limits the ability to explore dose-dependent effects and precludes direct comparison with standardized regimens such as those used in randomized trials. Data on antiviral therapies were also not uniformly available across participating sites and could not be reliably incorporated into adjusted analyses, precluding assessment of their potential impact on lymphocyte dynamics and outcomes. Lastly, our data reflect the COVID-19 era, and thus, extrapolation to non-COVID sepsis should be done with caution, though we emphasize that the underlying immunopathological mechanisms are likely to share significant overlap.

## Conclusion

In this study, Lymphopenia was common in hospitalized COVID-19 patients and independently associated with worse clinical outcomes, with persistent or worsening lymphopenia identifying those at highest risk. Monitoring lymphocyte dynamics provides an accessible window into immune function and prognosis, particularly in resource-limited settings. Furthermore, corticosteroid exposure was associated with impaired lymphocyte recovery, urging the need for a more nuanced, immune-informed approach to immunomodulatory therapies, especially in patients with signs of adaptive immune suppression. These findings support immune-informed management strategies in sepsis and severe viral infections and highlight the potential for personalized therapeutic approaches based on immune trajectory.

## Supplementary Information

Supplementary file 1.

## Data Availability

The data that underpin this analysis are highly detailed clinical data on individuals hospitalised with COVID-19. Due to the sensitive nature of this data and the associated privacy concerns, they are available via a governed data access mechanism following review of a data access committee. Data can be requested via the IDDO COVID-19 Data Sharing Platform (http://www.iddo.org/covid-19). The Data Access Application, Terms of Access and details of the Data Access Committee are available on the website. Briefly, the requirements for access are a request from a qualified researcher working with a legal entity who has a health and/or research remit; a scientifically valid reason for data access which adheres to appropriate ethical principles. The full terms are at: https://www.iddo.org/document/covid-19-data-access-guidelines. A small subset of sites who contributed data to this analysis have not agreed to pooled data sharing as above. In the case of requesting access to these data, please contact the corresponding author in the first instance who will look to facilitate access.

## Abbreviations

ISARIC-WHO: International Severe Acute Respiratory and emerging Infection Consortium
SARS-CoV-2: Severe acute respiratory syndrome coronavirus 2
COVID-19: Coronavirus disease 2019
OR: Odds-ratio
HR: Hazard-ratio
CI: Confidence interval
RT-PCR: Reverse transcription-polymerase chain reaction
ESM: Electronic supplementary material
eCRFs: Electronic case report forms
ICU: Intensive care unit
LCMM: Latent class mixed model
mHLA-DR: Monocytic human leukocyte antigen-DR
PD-1: Programmed cell death protein 1
LPS: Lipopolysaccharide
