# Supplementary material for: Immune dysregulation through longitudinal lymphocyte trajectories and their clinical determinants in hospitalized COVID-19 patients
Source: Intensive Care Med Exp. 2026 Feb 6;14:14. doi: 10.1186/s40635-026-00864-x (PMC12881241; doi:10.1186/s40635-026-00864-x)
Supplement: Supplementary file 1 [file 40635_2026_864_MOESM1_ESM.docx]

**Electronic Supplementary Material (ESM)**

Title: Immune Dysregulation through Longitudinal Lymphocyte Trajectories and Their Clinical Determinants in Hospitalized COVID-19 Patients

*José Pedro Cidade^1,2^, Fabio Silvio Taccone^3^, Luis Felipe Reyes^4,5^, Laura Merson^5,6^, Benjamin Lefevre^7^,  Barbara Wanjiru Citarella^6^, Arie Zainul fatoni^8^, Pedro Póvoa^1,2,9^, and the ISARIC Characterization Group

**1. Data source and governance**

Data were collected within the International Severe Acute Respiratory and Emerging Infection Consortium (ISARIC) – World Health Organization (WHO) Clinical Characterization Protocol (CCP) for Severe Emerging Infections. The standardized data collection framework, case report forms (CRFs), and operational manuals are publicly available on the ISARIC website (<https://isaric.org>). Data were captured via REDCap (Research Electronic Data Capture, Vanderbilt University) or equivalent locally hosted systems and harmonized for centralized curation by the ISARIC Data Platform hosted at the University of Oxford. Investigators retained full ownership of their local data.

**2. Inclusion and exclusion criteria**

Adult patients (≥18 years) hospitalized with confirmed SARS-CoV-2 infection were eligible for inclusion. Infection was defined by compatible clinical and radiological features and/or a positive RT-PCR test from a respiratory specimen. Patients were required to have available data on both absolute lymphocyte and total leukocyte counts during hospitalization.

To ensure meaningful characterization of lymphocyte dynamics, only patients with at least four absolute lymphocyte count measurements within the first 28 days of hospitalization were included. These measurements were required to include days 0, 1, 3, and 7, chosen to capture the critical early immune phase of sepsis and COVID-19, when lymphocyte depletion and early recovery typically occur. This time frame balances biological relevance with data availability across sites, ensuring comparability and robustness of early kinetic modeling.

Patients with more than 30% missing data in essential analytic variables, implausible laboratory values, or missing outcome data were excluded. Assessment of data completeness was performed on a prespecified set of essential analytic variables (see list). Patients with >30% missing in this essential variable set were excluded. For longitudinal inclusion, patients were required to have at least four absolute lymphocyte measurements within the first 28 days, including day 0, day 1, day 3 and day 7. Variables with >30% missingness were excluded from multivariable models unless clinically essential; variables with 10–30% missingness were considered for multiple imputation under a Missing At Random assumption in sensitivity analyses.

(List)

Demographics

- Age (years)
- Gender / Sex (male %)
- Height (cm) — supporting if BMI is needed;
- Weight (kg) — same as height

C. Key laboratory variables (ESSENTIAL)

- Absolute lymphocyte count (all available timepoints; specifically: day 0, day 1, day 3, day 7)
- Total leukocyte (white cell) count (all available timepoints)

G. Treatments / organ support (ESSENTIAL)

- Corticosteroid treatment (binary + timing/duration; time-varying if possible)
- Mechanical ventilation (invasive) — dates / duration
- Non-invasive ventilation — dates / duration
- Vasopressors / inotropes — indicator and dates/duration
- Renal replacement therapy — indicator and dates/duration
- High-flow nasal cannula (HFNC) — dates/duration
- ECMO — dates/duration
- Prone ventilation — dates/duration

H. Main outcomes (ESSENTIAL)

- ICU admission (yes/no; date if available)
- Duration of invasive mechanical ventilation, days (median [IQR])
- Duration of prone ventilation, days
- Duration of HFNC, days
- Duration of ECMO, days
- Time from admission to ICU, days
- Time from admission to IMV, days
- Duration of ICU stay (days)
- Duration of hospital stay (days)
- In-hospital outcome: Death / Discharge / Ongoing care / Transferred
- Primary outcome for manuscript: 28-day in-hospital mortality (explicit variable)

I. Other (ESSENTIAL / SUPPORTING)

- Country income classification (World Bank) — supporting but recommended as ESSENTIAL for international adjustments
- RT-PCR result / confirmation method (ESSENTIAL to define cohort)
- Dates of lab draws if available (essential for longitudinal mapping)

Censoring due to discharge or death before day 28 was handled by including all available measurements up to the censoring event, thus preventing informative dropout bias in longitudinal analyses.

**3. Data completeness and handling of missing data**

Data completeness was assessed for all key variables. Variables with more than 30% missingness were excluded from the main analysis unless clinically essential. Missing laboratory values within individuals were not imputed in the descriptive or survival analyses. In longitudinal and joint models, missing lymphocyte values were handled under the Missing At Random (MAR) assumption, implicitly accounted for by the mixed-effects modeling structure. Sensitivity analyses excluding patients with incomplete data produced consistent results.

**4. Variable definitions**

- Lymphopenia: defined as any absolute lymphocyte count <1×10⁹/L during hospitalization.
- Non-lymphopenia group: patients with no lymphocyte values below this threshold.
- Organ support interventions: included invasive and non-invasive mechanical ventilation, vasopressors/inotropes, and renal replacement therapy.
- Corticosteroid treatment: defined as any documented systemic corticosteroid administration during hospitalization, irrespective of agent, dose, duration, or specific treatment regimen
- The 28-day all-cause in-hospital mortality reported in the manuscript refers to deaths occurring during hospitalization within the first 28 days of admission. Deaths that occurred after hospital discharge were not captured in this dataset, as post-discharge follow-up was not uniformly available across centers. Patients discharged prior to day 28 were considered alive at discharge for the purpose of the 28-day in-hospital mortality outcome.
- Outcomes: The primary outcome was 28-day in-hospital all-cause mortality. Secondary outcomes included ICU admission, need for advanced organ support, hospital mortality, and the association between corticosteroid treatment and lymphocyte recovery trajectories.

**5. Statistical analysis details**

Continuous variables were summarized as mean (SD) or median (IQR), as appropriate. Categorical variables were reported as counts and percentages. Between-group comparisons were performed using the chi-square, t-test, or Kruskal–Wallis test.

**5.1 Survival analysis**

Kaplan–Meier survival curves were constructed to compare 28-day survival between lymphopenia-defined groups, using the log-rank test for univariate comparison. Multivariable Cox proportional hazards models were adjusted for age, sex, corticosteroid therapy, and organ support requirements. Proportional hazards assumptions were verified by Schoenfeld residuals.

**5.2 Multistate modeling**

Transitions between non-lymphopenic, lymphopenic, and death states were analyzed using a continuous-time, time-homogeneous Markov model with Aalen–Johansen estimation. This allowed for simultaneous estimation of competing transitions while accommodating intermediate states. Models were adjusted for age, sex, and corticosteroid therapy.

**5.3 Joint modeling of longitudinal and survival data**

To jointly model lymphocyte kinetics and mortality risk, a joint model combining a linear mixed-effects submodel for longitudinal lymphocyte counts and a Cox proportional hazards submodel for survival was applied using the JMbayes2 package in R. The longitudinal submodel included random intercepts and slopes for each patient, allowing for heterogeneity in baseline levels and rates of change. The survival submodel linked individual predicted lymphocyte trajectories to mortality risk.
Corticosteroid treatment was incorporated as a time-varying covariate.Models were adjusted for age, sex, and organ support variables.

**5.4 Sensitivity analyses**

Several sensitivity analyses were conducted:

1. Restricting the population to patients with complete 28-day follow-up.
2. Excluding patients who died within the first 7 days to account for early mortality bias.
3. Stratifying by corticosteroid exposure.
4. Comparing results using alternative lymphopenia thresholds (e.g., <0.8×10⁹/L).

**5.5 Statistical software**

All analyses were performed in R version 4.4.0 (R Foundation for Statistical Computing, Vienna, Austria) using the following packages: survival, lme4, JMbayes2, and msm. A two-sided p-value < 0.05 was considered statistically significant.

**Figures ESM**


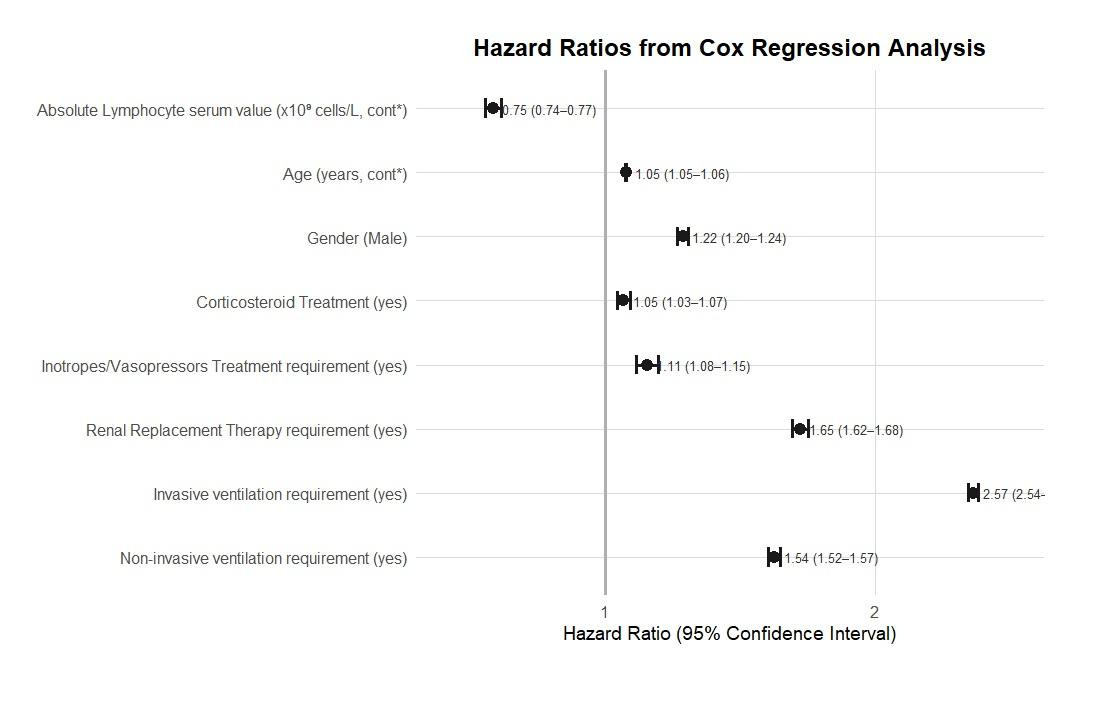


**Figure 1 ESM.** Forest plot of the Hazard ratios of in-hospital all-cause mortality from Cox regression analysis

*Cont stands for continuous variable


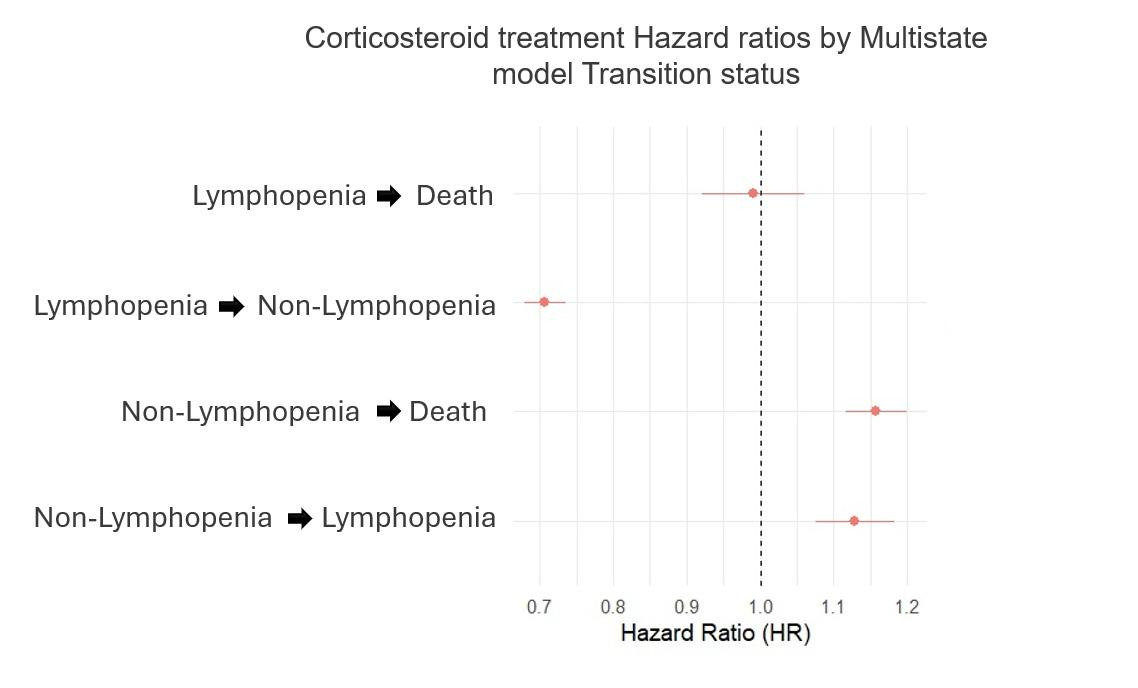


**Figure 2ESM.** Forest plot of the Hazard ratios of transition between states of Non-Lymphopenia, Lymphopenia and Death, with corticosteroid therapy

**ISARIC Clinical Characterisation Group**

Sabriya Abdalasalam, Alaa Abdalfattah Abdalhadi, Naana Reyam Abdalla, Almthani Hamza Abdalrheem, Saedah Abdeewi, Esraa Hassan Abdelgaum, Mohamed Abdelhalim, Mohammed Abdelkabir, Sheryl Ann Abdukahil, Lamees Adil Abdulbaqi, Nurul Najmee Abdulkadir, Eman Abdulwahed, Rawad Abdunabi, Ryuzo Abe, Laurent Abel, Ahmed Mohammed Abodina, Amal Abrous, Kamal Abu Jabal, Nashat Abu Salah, Abdurraouf Abusalama, Subhash Acharya, Andrew Acker, Safia Adem, Manuella Ademnou, Neill KJ Adhikari, Samuel Yaw Adu, Anthony Afum-Adjei Awuah, Melvin Agbogbatey, Saleh Al Ageel, Musaab Mohammed Ahmed, Aya Mustafa Ahmed, Zainab Ahmed Alaraji, Abdulrahman Ahmed Elhefnawy Enan, Reham Abdelhamid Ahmed Khalil, Ali Mostafa Ahmed Mohamed Abdelaziz, Kate Ainscough, Eka Airlangga, Tharwat Aisa, Ali Ait Hssain, Takako Akimoto, Ernita Akmal, Chika Akwani, Eman Al Qasim, Ahmed Alajeeli, Razi Alalqam, Zinah A. Alaraji, Safa Albatni, Angela Alberti, Tala Al-dabbous, Abdulkarim Aldoukali, Marta Alessi, Beatrice Alex, Kévin Alexandre, Abdulrahman Al-Fares, Asil Alflite, Huda Alfoudri, Qamrah Alhadad, Hoda Salem Alhaddad, Maali Khalid Mohamed Abdalla Alhasan, Hasan Alhouri, Ahmad Nabil Alhouri, Maha TagElser Mohammed Ali, Imran Ali, Yomna Ali Abdelghafar, Kazali Enagnon Alidjnou, Mahmoud Aljadi, Sarah Aljamal, Mohammed Alkahlout, Akram Alkaseek, Qabas Alkhafajee, Clotilde Allavena, Nathalie Allou, Abdulrahman Almjersah, Walaa Alrfaea, Moayad Alrifaee, Yousef Al-Saba'a, Entisar Alshareea, João Alves, João Melo Alves, Rita Alves, Joana Alves Cabrita, Maria Amaral, Amro Essam Amer, Nur Amira, Heidi Ammerlaan, Amos Amoako Adusei, John Amuasi, Roberto Andini, Claire Andrejak, Andrea Angheben, François Angoulvant, Séverine Ansart, Massimo Antonelli, Ardiyan Apriyana, Yaseen Arabi, Irene Aragao, Francisco Arancibia, Antonio Arcadipane, Patrick Archambault, Lukas Arenz, Jean-Benoît Arlet, Christel Arnold-Day, Lovkesh Arora, Elise Artaud-Macari, Diptesh Aryal, Motohiro Asaki, Angel Asensio, Elizabeth A. Ashley, Muhammad Ashraf, Muhammad Sheharyar Ashraf, Abir Ben Ashur, Franklin Asiedu-Bekoe, Namra Asif, Mohammad Asim, Grace Assi, Jean Baptiste Assie, Amirul Asyraf, Ahmed Atia, Minahel Atif, Asia Atif Abdelrhman Abdallahrs, Anika Atique, Moad Atlowly, Johann Auchabie, Hugues Aumaitre, Adrien Auvet, Ared Ayad, Ahmed Ayman Hassan Helmi, Laurène Azemar, Mohammed Azizeldin, Cecile Azoulay, Benjamin Bach, Antoine Bachelard, Delphine Bachelet, John Kenneth Baillie, J Kevin Baird, Erica Bak, Agamemnon Bakakos, Nazreen Abu Bakar, Hibah Bileid Bakeer, Ashraf Bakri, Andriy Bal, Mohanaprasanth Balakrishnan, Alessandra Bandera, Firouzé Bani-Sadr, Nicholas Yuri Barbosa, Wendy S. Barclay, Saef Umar Barnett, Michaela Barnikel, Cleide Barrigoto, Marie Bartoli, Cheryl Bartone, Joaquín Baruch, Romain Basmaci, Muhammad Fadhli Hassin Basri, AbdAlkarim Batool, Denise Battaglini, Jules Bauer, Diego Fernando Bautista Rincon, Alexandra Bedossa, Ker Hong Bee, Husna Begum, Aleksandr Beljantsev, Beatriz Amorim Beltrão, Marine Beluze, Nicolas Benech, Lionel Eric Benjiman, Suzanne Bennett, Luís Bento, Jan-Erik Berdal, Delphine Bergeaud, José Luis Bernal Sobrino, Giulia Bertoli, Lorenzo Bertolino, Simon Bessis, Adam Betz, Sybille Bevilcaqua, Karine Bezulier, Amar Bhatt, Claudia Bianco, Sandra Bichoka, Farah Nadiah Bidin, Felwa Bin Humaid, Mohd Nazlin Bin Kamarudin, Muhannud Binnawara, Patrick Biston, Laurent Bitker, Pablo Blanco-Schweizer, Catherine Blier, Frank Bloos, Mathieu Blot, Filomena Boccia, Laetitia Bodenes, Debby Bogaert, Anne-Hélène Boivin, Ariel Bolanga, Isabela Bolaños, Pierre-Adrien Bolze, Aurelius Bonifasius, Joseph Bonney, Diogo Borges, Raphaël Borie, Elisabeth Botelho-Nevers, Lila Bouadma, Yasmine Bouaraba, Olivier Bouchaud, Sabelline Bouchez, Kévin Bouiller, Laurence Bouillet, Camile Bouisse, Latsaniphone Bountthasavong, Anne-Sophie Boureau, John Bourke, Maude Bouscambert, Aurore Bousquet, Marielle Boyer-Besseyre, Maria Boylan, Fernando Augusto Bozza, Axelle Braconnier, Luca Brazzi, Patrick Breen, David Brewster, Kathy Brickell, Tessa Broadley, Petra Bryda, Nina Buchtele, Polina Bugaeva, Marielle Buisson, Erlina Burhan, Donald Buri, Aidan Burrell, Ingrid G. Bustos, Denis Butnaru, André Cabie, Susana Cabral, Joana Cabrita, Eder Caceres, Cyril Cadoz, Rui Caetano Garcês, Mia Callahan, Jose Andres Calvache, João Camões, Paul Campbell, Josie Campisi, Cecilia Canepa, Mireia Cantero, Janice Caoili, Pauline Caraux-Paz, Filipa Cardoso, Sofia Cardoso, Nelson Cardoso, Filipe Cardoso, Simone Carelli, Nicolas Carlier, Thierry Carmoi, Gayle Carney, Inês Carqueja, Marie-Christine Carret, François Martin Carrier, Ida Carroll, Leonor Carvalho, Maire-Laure Casanova, Mariana Cascão, Siobhan Casey, José Casimiro, Bailey Cassandra, Silvia Castañeda, Guylaine Castor-Alexandre, Ana Catarino, François-Xavier Catherine, Roberto Cauda, Giulio Giovanni Cavalli, Alexandros Cavayas, Adrian Ceccato, Ferruccio Ceriotti, Shelby Cerkovnik, Minerva Cervantes, Minerva Cervantes-Gonzalez, Muge Cevik, Anissa Chair, Catherine Chakveatze, Roberto Chalela, Adrienne Chan, Meera Chand, Jean-Marc Chapplain, Charlotte Charpentier, Julie Chas, Muhammad Mobin Chaudry, Jonathan Samuel Chávez Iñiguez, Anjellica Chen, Matthew Pellan Cheng, Antoine Cheret, Thibault Chiarabini, Julian Chica, Suresh Kumar Chidambaram, Leong Chin Tho, Catherine Chirouze, Hwa Jin Cho, Danoy Chommanam, Marie-Charlotte Chopin, Ting Soo Chow, Nathaniel Christy, Hiu Jian Chua, Jonathan Chua, José Miguel Cisneros Herreros, Barbara Wanjiru Citarella, Anna Ciullo, Jennifer Clarke, Rolando Claure-Del Granado, Sara Clohisey, Peter David Coakley, Caitriona Cody, Megan Coles, Jennifer Coles, Gwenhaël Colin, Michael Collins, Pamela Combs, Jennifer Connolly, Marie Connor, Anne Conrad, Elaine Conway, Graham S. Cooke, Hugues Cordel, Amanda Corley, Sabine Cornelis, Arianne Joy Corpuz, Andrea Cortegiani, Grégory Corvaisier, Aoife Cotter, Sandrine Couffin-Cadiergues, Roxane Courtois, Stéphanie Cousse, Juthaporn Cowan, Rachel Cregan, Charles Crepy D'Orleans, Cosimo Cristella, Gloria Crowl, Jonathan Crump, Claudina Cruz, Juan Luis Cruz Bermúdez, Jaime Cruz Rojo, Marc Csete, Ailbhe Cullen, Matthew Cummings, Gerard Curley, Elodie Curlier, Colleen Curran, Ana da Silva Filipe, Charlene Da Silveira, Al-Awwab Dabaliz, Andrew Dagens, Darren Dahly, Peter Daley, Zaina Dalloul, Jo Dalton, Heidi Dalton, Seamus Daly, Juliana Damas, Joycelyn Dame, Cammandji Damien, Nick Daneman, Jorge Dantas, Menno de Jong, Fernando De La Calle Prieto, Gillian de Loughry, Diego de Mendoza, Etienne De Montmollin, Ana Isabel de Pinho Oliveira, Rosanna De Rosa, Thushan de Silva, Peter de Vries, Alexa Debard, Bianca DeBenedictis, Marie-Pierre Debray, Nathalie DeCastro, William Dechert, Romain Decours, Eve Defous, Isabelle Delacroix, Alexandre Delamou, Karen Delavigne, Nathalie M. Delfos, Andrea Dell'Amore, Christelle Delmas, Pierre Delobel, Ismaila Deme, Elisa Demonchy, Emmanuelle Denis, Dominique Deplanque, Pieter Depuydt, Diane Descamps, Mathilde Desvallées, Santi Dewayanti, Pathik Dhangar, Alpha Diallo, Souleymane Taran Diallo, Sylvain Diamantis, Andrea Dias, André Dias, Juan Jose Diaz, Priscila Diaz, Rodrigo Diaz, Kévin Didier, Jean-Luc Diehl, Aurélien Dinh, Alphonsine Diouf, Yael Dishon, Cedric Djadda, Félix Djossou, Nikita Dobremel, Annemarie B. Docherty, Helen Doherty, Arjen M Dondorp, Christl A. Donnelly, Yoann Donohue, Sean Donohue, Peter Doran, Céline Dorival, Eric D'Ortenzio, Phouvieng Douangdala, James Joshua Douglas, Nathalie Dournon, Thomas Drake, Aoife Driscoll, Ibrahim Kwaku Duah, Vincent Dubee, François Dubos, Audrey Dubot-Pérès, Alexandre Ducancelle, Susanne Dudman, Abhijit Duggal, Paul Dunand, Jake Dunning, Mathilde Duplaix, Emanuele Durante-Mangoni, Lucian Durham III, Bertrand Dussol, Xavier Duval, Anne Margarita Dyrhol-Riise, Sim Choon Ean, Marco Echeverria-Villalobos, Michael Edelstein, Khadeja Ehzaz, Carla Eira, Mohammed El Sanharawi, Subbarao Elapavaluru, Mohammad Elbahnasawy, Brigitte Elharrar, Hamida ELMagrahi, Lauren Eloundou, Philippine Eloy, Tarek Elshazly, Wafa Elsokni, Aml Ahmed Eltayeb, Iqbal Elyazar, Zarief Kamel Emad, Hussein Embarek, Isabelle Enderle, Tomoyuki Endo, Gervais Eneli, Chan Chee Eng, Ilka Engelmann, Vincent Enouf, Olivier Epaulard, Haneen Esaadi, Mariano Esperatti, Catarina Espírito Santo, Marina Esposito-Farese, Rachel Essaka, João Estevão, Manuel Etienne, Anna Greti Everding, Mirjam Evers, Marc Fabre, Isabelle Fabre, Asgad Osman Abdalla Fadlalla, Amna Faheem, Arabella Fahy, Cameron J. Fairfield, Komal Fareed, Pedro Faria, Ahmed Farooq, Hanan Fateena, Salem Fatima, Arie Zainul Fatoni, Karine Faure, Raphaël Favory, Mohamed Fayed, Niamh Feely, Eoin Feeney, Susana Fernandes, Jorge Fernandes, Marília Andreia Fernandes, François-Xavier Ferrand, Joana Ferrão, Mário Ferraz, Isabel Ferreira, Sílvia Ferreira, Benigno Ferreira, Bernardo Ferreira, Nicolas Ferriere, Valentina Ferroni, Céline Ficko, Thomas Flament, Tom Fletcher, Aline-Marie Florence, Letizia Lucia Florio, Deirdre Flynn, Jean Foley, Victor Fomin, Patricia Fontela, Simon Forsyth, Denise Foster, Giuseppe Foti, Berline Fotso, Erwan Fourn, Robert A. Fowler, Marianne Fraher, Diego Franch-Llasat, Pierre Frange, John F. Fraser, Christophe Fraser, Ricardo Fritz, Stéphanie Fry, Nora Fuentes, Argin G, Valérie Gaborieau, Rostane Gaci, Massimo Gagliardi, Jean-Charles Gagnard, Amandine Gagneux-Brunon, Sérgio Gaião, Linda Gail Skeie, Adham Mohamed Galal Mohamed Ramadan, Phil Gallagher, Carrol Gamble, Yasmin Gani, Arthur Garan, Rebekha Garcia, Noelia García Barrio, Julio Garcia Rodriguez, Julia Garcia-Diaz, Esteban Garcia-Gallo, Denis Garot, Valérie Garrait, Anatoliy Gavrylov, Alexandre Gaymard, Johannes Gebauer, Eva Geraud, Louis Gerbaud Morlaes, Nuno Germano, Malak Ghemmeid, Praveen Kumar Ghisulal, Jade Ghosn, Marco Giani, Carlo Giaquinto, Séverine Gibowski, Tristan Gigante, Guillermo Giordano, Michelle Girvan, Valérie Gissot, Gezy Giwangkancana, Daniel Glikman, Petr Glybochko, Eric Gnall, Geraldine Goco, François Goehringer, Siri Goepel, Jean-Christophe Goffard, Jin Yi Goh, Brigitta Golács, Jonathan Golob, Rui Gomes, Joan Gómez-Junyent, Marie Gominet, Alicia Gonzalez, Patricia Gordon, Isabelle Gorenne, Laure Goubert, Cécile Goujard, Tiphaine Goulenok, Margarite Grable, Jeronimo Graf, Edward Wilson Grandin, Pascal Granier, Giacomo Grasselli, Christopher A. Green, William Greenhalf, Segolène Greffe, Domenico Luca Grieco, Matthew Griffee, Fiona Griffiths, Ioana Grigoras, Albert Groenendijk, Fassou Mathias Grovogui, Heidi Gruner, Yusing Gu, Jérémie Guedj, Martin Guego, Anne-Marie Guerguerian, Daniela Guerreiro, Romain Guery, Anne Guillaumot, Laurent Guilleminault, Thomas Guimard, Daniel Haber, Ali Hachemi, Abdurrahman Haddud, Nadir Hadri, Wael Hafez, Fakhir Raza Haidri, Fatima Mhd Rida Hajij, Sheeba Hakak, Matthew Hall, Sophie Halpin, Shaher Hamdan, Abdelhafeez Hamdi, Ansley Hamer, Raph L. Hamers, Rebecca Hamidfar, Naomi Hammond, Terese Hammond, Lim Yuen Han, Matly Hanan, Rashan Haniffa, Kok Wei Hao, Hayley Hardwick, Ewen M. Harrison, Alan Hartman, Mohd Shahnaz Hasan, Mohammad Ali Nabil Hasan, Sulieman Hasan, Madiha Hashmi, Junaid Hashmi, Amoni Hassan, Ebtisam Hassanin, Muhammad Hayat, Ailbhe Hayes, Leanne Hays, Lars Heggelund, Ahmed Helmi, Ross Hendry, Martina Hennessy, Aquiles Rodrigo Henriquez-Trujillo, Maxime Hentzien, Diana Hernandez, Andrew Hershey, Astarini Hidayah, Eibhlin Higgins, Samuel Hinton, Hiroaki Hiraiwa, Maya Hites, Hikombo Hitoto, Yi Bin Ho, Antonia Ho, Alexandre Hoctin, Isabelle Hoffmann, Wei Han Hoh, Oscar Hoiting, Jan Cato Holter, Peter Horby, Juan Pablo Horcajada, Mabrouka Houderi, Stuart Houltham, Jimmy Ming-Yang Hsu, Jean-Sébastien Hulot, Abby Hurd, Iqbal Hussain, Aliae Mohamed Hussein, Mahmood Hussein, Fatima Ibrahim, Bashir Ibran, Samreen Ijaz, M. Arfan Ikram, Patrick Imbert, Rana Imran Sikander, Hugo Inácio, Carmen Infante Dominguez, Yun Sii Ing, Mariachiara Ippolito, Vera Irawany, Sarah Isgett, Tiago Isidoro, Nadiah Ismail, Margaux Isnard, Junji Itai, Danielle Jaafar, Salma Jaafoura, Hamza Jaber, Julien Jabot, Clare Jackson, Victoria Janes, Stéphane Jaureguiberry, Jeffrey Javidfar, Denise Jaworsky, Florence Jego, Anilawati Mat Jelani, Synne Jenum, Ruth Jimbo-Sotomayor, Ong Yiaw Joe, Ruth Noemí Jorge García, Cédric Joseph, Mark Joseph, Evelyn Joson, Mercé Jourdain, Philippe Jouvet, Anna Jung, Dafsah Juzar, Ouifiya Kafif, Florentia Kaguelidou, Neerusha Kaisbain, Sabrina Kali, Smaragdi Kalomoiri, Muhammad Aisar Ayadi Kamaluddin, Armand Saloun Kamano, Zul Amali Che Kamaruddin, Nadiah Kamarudin, Darshana Hewa Kandamby, Kong Yeow Kang, Dyah Kanyawati, Mohamed Karghul, Pratap Karpayah, Christiana Kartsonaki, Daisuke Kasugai, Kevin Katz, Christy Kay, Lamees Kayyali, Seán Keating, Aoife Kelly, Niamh Kelly, Sadie Kelly, Yvelynne Kelly, Maeve Kelsey, Sommay Keomany, Maeve Kernan, Younes Kerroumi, Sharma Keshav, Shams Khail, Sarah Khaled, Imrana Khalid, Zineb Khalil, Antoine Khalil, Irfan Khan, Sushil Khanal, Michelle E. Kho, Denisa Khoo, Saye Khoo, Ryan Khoo, Muhammad Nasir Khoso, Khor How Kiat, Yuri Kida, Peter Kiiza, Beathe Kiland Granerud, Anders Benjamin Kildal, Jae Burm Kim, Antoine Kimmoun, Paul Klenerman, Gry Kloumann Bekken, Stephen R Knight, Robin Kobbe, Chamira Kodippily, Sabin Koirala, Stephanie Kouba, Karifa Kourouma, Mohamed Lamine Kourouma, Karolina Krawczyk, Ali Kredan, Vinothini Krishnan, Sudhir Krishnan, Oksana Kruglova, Anneli Krund, Pei Xuan Kuan, Ganesh Kumar, Deepali Kumar, Dinesh Kuriakose, Ethan Kurtzman, Demetrios Kutsogiannis, Galyna Kutsyna, Sylvie Kwedi, Konstantinos Kyriakoulis, Marie Lachatre, Karine Lacombe, Marie Lacoste, John G. Laffey, Nadhem Lafhej, Marie Lagrange, Fabrice Laine, Olivier Lairez, Antonio Lalueza, Marc Lambert, Marie Langelot-Richard, Vincent Langlois, Cédric Laouénan, Samira Laribi, Delphine Lariviere, Jamaica Laroza, Stéphane Lasry, Youssef Latifeh, Odile Launay, Didier Laureillard, Yoan Lavie-Badie, Andy Law, Teresa Lawrence, Minh Le, Clément Le Bihan, Cyril Le Bris, Georges Le Falher, Lucie Le Fevre, Quentin Le Hingrat, Marion Le Maréchal, Soizic Le Mestre, Guillaume Le Meut, Gwenaël Le Moal, Vincent Le Moing, Hervé Le Nagard, Ema Leal, Marta Leal Santos, Yi Lin Lee, Heng Gee Lee, James Lee, Biing Horng Lee, Todd C. Lee, Gary Leeming, Bénédicte Lefebvre, Laurent Lefebvre, Benjamin Lefèvre, Sylvie LeGac, Merili-Helen Lehiste, Jean-Daniel Lelievre, François Lellouche, Adrien Lemaignen, Véronique Lemee, Anthony Lemeur, Gretchen Lemmink, Ha Sha Lene, Jenny Lennon, Marc Leone, Tanel Lepik, Quentin Lepiller, François-Xavier Lescure, Mathieu Lesouhaitier, Andrew Letizia, Sophie Letrou, Bruno Levy, Yves Levy, Claire Levy-Marchal, Katarzyna Lewandowska, Gianluigi Li Bassi, Janet Liang, Geoffrey Liegeon, Wei Shen Lim, Kah Chuan Lim, Chantre Lima, Lim Lina, Bruno Lina, Andreas Lind, Guillaume Lingas, Sylvie Lion-Daolio, Keibun Liu, Marine Livrozet, Patricia Lizotte, Antonio Loforte, Navy Lolong, Leong Chee Loon, Diogo Lopes, Dalia Lopez-Colon, Jose W. López-Revilla, Anthony L. Loschner, Paul Loubet, Bouchra Loufti, Guillame Louis, Silvia Lourenco, Lee Lee Low, Jia Shyi Loy, Carlos Lumbreras Bermejo, Carlos M. Luna, Olguta Lungu, Miles Lunn, Liem Luong, Nestor Luque, Dominique Luton, Olavi Maasikas, Moïse Machado, Sara Machado, Gabriel Macheda, Claire Madelaine, Guillermo Maestro de la Calle, Rafael Mahieu, Sophie Mahy, Ana Raquel Maia, Lars S. Maier, Oumou Maiga Ascofare, Mylène Maillet, Nimisha Abdul Majeed, Maximilian Malfertheiner, Nadia Malik, Wajeeha Malik, Dayana Malla, Paddy Mallon, Fernando Maltez, Denis Malvy, Victoria Manda, Laurent Mandelbrot, Frank Manetta, Julie Mankikian, Edmund Manning, Aldric Manuel, Veronika Maráczi, Samuel Markowicz, Ana Marques, Megan Marshal, John Marshall, Dori-Ann Martin, Emily Martin, Guillaume Martin-Blondel, F. Eduardo Martinez, Ignacio Martin-Loeches, Martin Martinot, Alejandro Martín-Quiros, João Martins, Nuno Martins, Ana Martins, Gennaro Martucci, Olga Martynenko, Eva Miranda Marwali, Marsilla Marzukie, David Maslove, Sabina Mason, Sobia Masood, Moise Massoma, Palmer Masumbe, Mohd Basri Mat Nor, Moshe Matan, Christina Mathew, Mathieu Mattei, Laurence Maulin, Juergen May, Mayfong Mayxay, Thierry Mazzoni, Lisa Mc Sweeney, Colin McArthur, Peter McCanny, Aine McCarthy, Colin McCloskey, Rachael McConnochie, Sherry McDermott, Sarah E. McDonald, Natalie McEvoy, Allison McGeer, Kenneth A. McLean, Paul McNally, Bairbre McNicholas, Edel Meaney, Cécile Mear-Passard, Maggie Mechlin, Omar Mehkri, Ferruccio Mele, João João Mendes, Kusum Menon, France Mentré, Alexander J. Mentzer, Emmanuelle Mercier, Antoine Merckx, Mayka Mergeay-Fabre, Laura Merson, António Mesquita, Osama Metwally, Agnès Meybeck, Dan Meyer, Mehdi Mezidi, Isabelle Michelet, Efstathia Mihelis, Vladislav Mihnovit, Duha Milad Abdullah, Jennene Miller, Hugo Miranda-Maldonado, Nor Arisah Misnan, Tahira Jamal Mohamed, Nouralsabah Mohamed, Nik Nur Eliza Mohamed, Alaa Mohamed Ads, Ahmed Reda Mohamed Elsayed Abdelhalim, Shrouk Fawze Mohammed Mostafa, Omer Abdullah Mohammedelhassan, Saad A. Moharam, Diana Molino, Elena Molinos, Brenda Molloy, Geraldine Moloney, Mary Mone, Agostinho Monteiro, Claudia Montes, Giorgia Montrucchio, Sarah Moore, Shona C. Moore, Lina Morales Cely, Marwa Morgom, Lucia Moro, Ana Motos, Clara Mouton Perrot, Julien Moyet, Suleiman Haitham Mualla, Aisha Kalsoom Mufti, Ng Yong Muh, Mo'nes Muhaisen, Dzawani Muhamad, Jimmy Mullaert, Karl Erik Müller, Fredrik Müller, Daniel Munblit, Syed Muneeb Ali, Laveena Munshi, Aisling Murphy, Patrick Murray, Marlène Murris, Srinivas Murthy, Himed Musaab, Dana Mustafa, Dimitra Melia Myrodia, Farah Nadia Mohd-Hanafiah, Dave Nagpal, Blanka Nagybányai-Nagy, Herwin Nanda Boudoin, Mangala Narasimhan, Adel Gerges Nassif Metri, Nadège Neant, Coca Necsoi, Nikita Nekliudov, Matthew Nelder, Erni Juwita Nelwan, Emily Neumann, Wing Yiu Ng, Pauline Yeung Ng, Anthony Nghi, Duc Nguyen, Orna Ni Choileain, Niamh Ni Leathlobhair, Nerissa Niba, Alistair D Nichol, Nurul Amani Mohd Noordin, Nurul Faten Izzati Norharizam, Mahdad Noursadeghi, Adam Nowinski, Saad Nseir, Leonard Numfor, Nurnaningsih Nurnaningsih, Dwi Utomo Nusantara, Elsa Nyamankolly, Fionnuala O Brien, Annmarie O Callaghan, Annmarie O'Callaghan, Giovanna Occhipinti, Sarah O'Connell, Derbrenn OConnor, Max O'Donnell, Ebenezer Oduro-Mensah, Tawnya Ogston, Takayuki Ogura, Tak-Hyuk Oh, Sophie O'Halloran, Katie O'Hearn, Sally-Ann Ohene, Shinichiro Ohshimo, Agnieszka Oldakowska, João Oliveira, Joseph Oliver-Commey, Piero L. Olliaro, Inge Christoffer Olsen, Alsarrah Ali Mohammed Omer, Pierre Ondobo, David S. Y. Ong, Jee Yan Ong, Wilna Oosthuyzen, Peter Openshaw, Saijad Orakzai, Claudia Milena Orozco-Chamorro, Mohamed Osama Elsayed Soliman, Linda O'Shea, Miriam O'Sullivan, Siti Zubaidah Othman, Eman Othman, Rachida Ouissa, Christian Owoo, Micheal Owusu, Ama Akyampomaa Owusu-Asare, Eric Oziol, Patricia Pacheco, Maïder Pagadoy, Justine Pages, Massimo Palmarini, Carlo Palmieri, Giovanna Panarello, Prasan Kumar Panda, Hem Paneru, Lai Hui Pang, Mauro Panigada, Nathalie Pansu, Aurélie Papadopoulos, Rachael Parke, Melissa Parker, Bruno Pastene, Fabian Patauner, Mohan Dass Pathmanathan, Luís Patrão, Patricia Patricio, Lisa Patterson, Christelle Paul, Mical Paul, Jorge Paulos, William A. Paxton, Jean-François Payen, Sandra L Peake, Kalaiarasu Peariasamy, Miguel Pedrera Jiménez, Giles J. Peek, Florent Peelman, Nathan Peiffer-Smadja, Vincent Peigne, Mare Pejkovska, Paolo Pelosi, Rui Pereira, Daniel Perez, Thomas Perpoint, Antonio Pesenti, Lenina Pessey, Vincent Pestre, Lenka Petrou, Michele Petrovic, Ventzislava Petrov-Sanchez, Gilles Peytavin, Richard Odame Philips, Ooyanong Phonemixay, Soulichanya Phoutthavong, Michael Piagnerelli, Gilles Pialoux, Olivier Picone, Maria de Piero, Carlos Pimentel, Raquel Pinto, Catarina Pires, Lionel Piroth, Ayodhia Pitaloka, Chiara Piubelli, Riinu Pius, Simone Piva, Laurent Plantier, Hon Shen Png, Julien Poissy, Ryadh Pokeerbux, Maria Pokorska-Spiewak, Sergio Poli, Georgios Pollakis, Diane Ponscarme, Jolanta Popielska, Diego Bastos Porto, Andra-Maris Post, Douwe F. Postma, Valérie Pourcher, Diana Póvoas, Jeff Powis, Sofia Prapa, Viladeth Praphasiri, Sébastien Preau, Christian Prebensen, Jean-Charles Preiser, Anton Prinssen, Gamage Dona Dilanthi Priyadarshani, Lucia Proença, Sravya Pudota, Bambang Pujo Semedi, Peter Puplampu, Gregory Purcell, Luisa Quesada, Víctor Quirós González, Else Quist-Paulsen, Mohammed Quraishi, Fadi Qutishat, Christian Rabaud, Ebenezer Rabindrarajan, Aldo Rafael, Marie Rafiq, Abdelrahman Ragab, Mutia Rahardjani, Rozanah Abd Rahman, Ahmad Kashfi Haji Ab Rahman, Fernando Rainieri, Giri Shan Rajahram, Pratheema Ramachandran, José Ramalho, Ahmad Afiq Ramli, Grazielle Viana Ramos, Muhammad Asim Rana, Rajavardhan Rangappa, Hervé Raoul, Christophe Rapp, Thalha Rashan, Aasiyah Rashan, Ghulam Rasheed, Menaldi Rasmin, Indrek Rätsep, Cornelius Rau, Tharmini Ravi, Andre Real, Stanislas Rebaudet, Sarah Redl, Brenda Reeve, Dag Henrik Reikvam, Renato Reis, Jonathan Remppis, Hongru Ren, Hanna Renk, Anne-Sophie Resseguier, Matthieu Revest, Oleksa Rewa, Luis Felipe Reyes, Maria Ines Ribeiro, Denise Richardson, David Richardson, Laurent Richier, Siti Nurul Atikah Ahmad Ridzuan, Asgar Rishu, Patrick Rispal, Karine Risso, Maria Angelica Rivera Nuñez, Chiara Robba, André Roberto, David L. Robertson, Olivier Robineau, Ferran Roche-Campo, Paola Rodari, Simão Rodeia, Bernhard Roessler, Claire Roger, Amanda Rojek, Roberto Roncon-Albuquerque Jr, Mélanie Roriz, Manuel Rosa-Calatrava, Michael Rose, Dorothea Rosenberger, Andrea Rossanese, Matteo Rossetti, Patrick Rossignol, Carine Roy, Benoît Roze, Desy Rusmawatiningtyas, Clark D. Russell, Maeve Ryan, Aleksander Rygh Holten, Isabela Saba, Sairah Sadaf, Musharaf Sadat, Valla Sahraei, Abdurraouf Said, Fodé Bangaly Sako, Moamen Salah, Ali Alaa Salah Eldin Mohamed Abbas, Nawal Salahuddin, Leonardo Salazar, Mohammed Saleh Alyasiri, Gabriele Sales, Charlotte Salmon Gandonniere, Hélène Salvator, Yehia Samir Shaaban Aly Orabi, Olivier Sanchez, Emely Sanchez, Angel Sanchez-Miralles, Gyan Sandhu, Zulfiqar Sandhu, Pierre-François Sandrine, Oana Săndulescu, Marlene Santos, Lurdes Santos, Shirley Sarfo-Mensah, Bruno Sarmento Banheiro, Benjamine Sarton, Sree Satyapriya, Rumaisah Satyawati, Yen Tsen Saw, Justin Schaffer, Tjard Schermer, Arnaud Scherpereel, Marion Schneider, János Schnur, Michael Schwameis, Gary Schwartz, Janet T. Scott, Nicholas Sedillot, Tamara Seitz, Mageswari Selvarajoo, Malcolm G. Semple, Rasidah Bt Senian, Eric Senneville, Claudia Sepulveda, Tânia Sequeira, Ary Serpa Neto, Pablo Serrano Balazote, Ellen Shadowitz, Syamin Asyraf Shahidan, Laila Shalabi, Haitam Shames, Anuraj Shankar, Shaikh Sharjeel, Pratima Sharma, Catherine A. Shaw, Victoria Shaw, John Robert Sheenan, Dr. Rajesh Mohan Shetty, Mohiuddin Shiekh, Nobuaki Shime, Keiki Shimizu, Hiroaki Shimizu, Sally Shrapnel, Hoi Ping Shum, Nassima Si Mohammed, Ng Yong Siang, Jeanne Sibiude, Bountoy Sibounheuang, Louise Sigfrid, Piret Sillaots, Maria Joao Silva, Rogério Silva, Catarina Silva, Benedict Sim Lim Heng, Wai Ching Sin, Punam Singh, Mahendra Singh, Pompini Agustina Sitompul, Karisha Sivam, Vegard Skogen, Benjamin Smood, Coilin Smyth, Dominic So, Tze Vee Soh, Tom Solomon, Joshua Solomon, Emily Somers, Agnès Sommet, Rima Song, Tae Song, Jack Song Chia, Albert Sotto, Edouard Soum, Ana Chora Sousa, Marta Sousa, Maria Sousa Uva, Vicente Souza-Dantas, Mamadou Saliou Sow, Alexandra Sperry, Elisabetta Spinuzza, B. P. Sanka Ruwan Sri Darshana, Shiranee Sriskandan, Sarah Stabler, Thomas Staudinger, Stephanie-Susanne Stecher, Trude Steinsvik, Ymkje Stienstra, Birgitte Stiksrud, Eva Stolz, Amy Stone, Adrian Streinu-Cercel, Anca Streinu-Cercel, David Stuart, Decy Subekti, Jacky Y. Suen, Prasanth Sukumar, Charlotte Summers, Dubravka Supic, Deepashankari Suppiah, Magdalena Surovcová, Atie Suwarti, Andrey Svistunov, Sarah Syahrin, Augustina Sylverken, Konstantinos Syrigos, Shirin Tabrizi, Fabio S. Taccone, Shahdattul Mawarni Taib, Ewa Talarek, Sara Taleb, Cheikh Talla, Jelmer Talsma, Maria Lawrensia Tampubolon, Yan Chyi Tan, Kim Keat Tan, Taku Tanaka, Huda Taqdees, Coralie Tardivon, Yousef Tarek Kamal Mostafa, Ali Tarhabat, Pierre Tattevin, M Azhari Taufik, Hassan Tawfik, Tze Yuan Tee, João Teixeira, Sze Kye Teoh, Vanessa Teotonio, François Téoulé, Olivier Terrier, Hubert Tessier-Grenier, Adrian Tey, Alif Adlan Mohd Thabit, Zhang Duan Tham, Suvintheran Thangavelu, Elmi Theron, Vincent Thibault, Simon-Djamel Thiberville, Benoît Thill, Jananee Thirumanickam, Shaun Thompson, Emma C. Thomson, David Thomson, Mathew Thorpe, Surain Raaj Thanga Thurai, Ryan S. Thwaites, Paul Tierney, Vadim Tieroshyn, Peter S Timashev, Jean-François Timsit, Noémie Tissot, Jordan Zhien Yang Toh, Maria Toki, Kristian Tonby, Sia Loong Tonnii, Marta Torre, Antoni Torres, Margarida Torres, Rosario Maria Torres Santos-Olmo, Hernando Torres-Zevallos, Aboubacar Tounkara, Michael Towers, Fodé Amara Traoré, Tony Trapani, Cécile Tromeur, Ioannis Trontzas, Jeanne Truong, Christelle Tual, Sarah Tubiana, Helen Tuite, Alexis F. Turgeon, Lance C.W. Turtle, Anders Tveita, Pawel Twardowski, Makoto Uchiyama, PG Ishara Udayanga, Andrew Udy, Roman Ullrich, Alberto Uribe, Asad Usman, Timothy M. Uyeki, Cristinava Vajdovics, Luís Val-Flores, Marcel van den Berge, Machteld van der Feltz, Peter Van der Voort, Sylvie Van Der Werf, Eric Van Gorp, Laura van Gulik, Jarne Van Hattem, Carolien van Netten, Frank van Someren Gréve, Gitte Van Twillert, Hugo Van Willigen, Noémie Vanel, Michael Varrone, Shoban Raj Vasudayan, Charline Vauchy, Shaminee Veeran, Aurélie Veislinger, Sara Ventura, Annelies Verbon, César Vieira, Deepak Vijayan, Judit Villar, Andrea Villoldo, Manivanh Vongsouvath, Fanny Vuotto, Suhaila Abdul Wahab, Noor Hidayu Wahab, Nadirah Abdul Wahid, Marina Wainstein, Laura Walsh, Steve Webb, Tan Pei Wen, Sanne Wesselius, Murray Wham, Bryan Whelan, Nicole White, Aurélie Wiedemann, Surya Otto Wijaya, Virginie Williams, Patricia J Williams, Bailey Williams, Evert-Jan Wils, Karolina Witt, Jessica Wittman, Xin Ci Wong, Yew Sing Wong, Teck Fung Wong, Calvin Wong, Lim Saio Xian, Ioannis Xynogalas, Siti Rohani Binti Mohd Yakop, Masaki Yamazaki, Elizabeth Yarad, Yazdan Yazdanpanah, Nicholas Yee Liang Hing, Abdelrahman Yehia Mahmoud Abdelaal, Cécile Yelnik, Chian Hui Yeoh, Touxiong Yiaye, Hodane Yonis, Obada Yousif, Saptadi Yuliarto, Akram Zaaqoq, Marion Zabbe, Masliza Zahid, Nor Zaila Binti Zaidan, Maria Zambon, Miguel Zambrano, Alberto Zanella, Konrad Zawadka, Nurul Zaynah, Hiba Zayyad, Alexander Zoufaly, David Zucman.
